# Unveiling the state of the art: a systematic review and meta-analysis of paper-based microfluidic devices

**DOI:** 10.3389/fbioe.2024.1421831

**Published:** 2024-08-21

**Authors:** Rodrigo García-Azuma, Karen Werner, Cristina Revilla-Monsalve, Oscar Trinidad, Nelly F. Altamirano-Bustamante, Myriam M. Altamirano-Bustamante

**Affiliations:** ^1^ Unidad de Investigación en Enfermedades Metabólicas, Centro Médico Nacional Siglo XXI, Instituto Mexicano del Seguro Social, Ciudad de México, Mexico; ^2^ Servicio de Endocrinología, Instituto Nacional de Pediatría, Ciudad de México, Mexico

**Keywords:** paper-based microfluidic device, immunoassays, point-of-care diagnostics, meta-analysis, resource-limited settings, diagnostic technologies

## Abstract

**Introduction:**

This systematic review and meta-analysis present a comprehensive evaluation of paper-based microfluidic devices, focusing on their applications in immunoassays. These devices are emerging as innovative solutions to democratize access to diagnostic technologies, especially in resource-limited settings. Our review consolidates findings from diverse studies to outline advancements in paper-based microfluidic technology, including design intricacies and operational efficacy. Key advantages such as low cost, portability, and ease of use are highlighted.

**Materials and Methods:**

The review categorizes literature based on the design and operational nuances of these diagnostic tools, exploring various methodologies, fabrication techniques, detection methods, and applications, particularly in protein science. The meta-analysis extends to the diverse applications of these technologies, providing a framework for classifying and stratifying their uses in diagnostics.

**Results and discussion:**

Notable findings include a critical analysis of performance metrics, such as sensitivity and specificity. The review addresses challenges, including the need for further validation and optimization for broader clinical applications. A critical discussion on the validation processes, including cross-validation and rigorous control testing, is provided to ensure the robustness of microfluidic devices. This study offers novel insights into the computational strategies underpinning these technologies and serves as a comprehensive roadmap for future research, potentially broadening the impact across the protein science universe.

## 1 Introduction

In recent years, the field of diagnostic technologies has witnessed a transformative shift towards the development of portable, cost-effective, and user-friendly devices (Liu et al., 2011; Tian et al., 2017; Uddin et al., 2021). Among these innovations, paper-based microfluidic devices have emerged as a revolutionary platform for conducting immunoassays, offering unparalleled advantages in terms of simplicity, accessibility, and resource efficiency (Menegatti et al., 2013) exemplified by the development of a plug-based microfluidic chip capable of performing agglutination assays for AB0 and D (Rh) blood typing.

This systematic review aims to provide a comprehensive overview of the current state of the art in paper-based microfluidic devices with a primary focus on their application in immunoassays. The urgency to improve global healthcare accessibility, especially in resource-limited settings, has fuelled the demand for point-of-care diagnostic tools (Zhu et al., 2018). Immunoassays, which detect the presence of specific biomolecules through the interaction with antibodies, play a pivotal role in diagnosing various diseases, monitoring treatment efficacy, and facilitating timely interventions (Li et al., 2023). However, traditional immunoassay platforms often face challenges related to cost, complexity, and the need for sophisticated instrumentation in controlled laboratory settings (Novo et al., 2014), as exemplified by the development of a point-of-care prototype that integrates capillary microfluidics with microfabricated photodiodes and electronic instrumentation, achieving comparable performance to traditional bench-top systems but requiring significantly simpler and more accessible setup. Paper-based microfluidic devices have gained attention in the field because they provide key advantages over traditional immunoassay platforms including the spontaneous and power-free fluid transport from the sample to the detection zone through capillary action, making them well suited for point-of-care testing due to their portability (Dixit et al., 2011; Sinha et al., 2022b). In comparison to alternative materials used in microfluidic devices, such as glass, polymers, or silicon, paper offers distinct advantages (Seia et al., 2014; Deng and Jiang, 2019). Notably, paper is inexpensive, widely available, biodegradable, and can be easily modified for specific applications (Lepowsky et al., 2017). Moreover, the simplicity of fabrication and the absence of complex microfabrication techniques contribute to the cost-effectiveness of paper-based devices, making them an attractive choice for resource-limited environments (Ahmed et al., 2016), as demonstrated by the use of wax printing methods to create hydrophobic barriers on paper, allowing for the rapid and inexpensive production of microfluidic channels. The review will delve into the various designs and configurations of paper-based immunoassays, emphasizing key features such as multiplexing capabilities, sensitivity, specificity, integration with detection methods among others through a meta-analysis for novel qualitative and quantitative results (Uddin et al., 2021). While paper-based microfluidic devices exhibit tremendous promise, there are challenges that need to be addressed to enhance their reliability and performance.

This systematic review and meta-analysis will delve into the multifaceted landscape of paper-based microfluidic devices in immunoassays, highlighting the remarkable strides made thus far, while also acknowledging the hurdles that lie ahead (Apilux et al., 2013), as evidenced by the development of automated paper-based devices for sequential multistep sandwich enzyme-linked immunosorbent assays (ELISAs) using inkjet printing, which simplifies the ELISA process and reduces reagent consumption while maintaining high sensitivity and specificity. The inherent advantages of microfluidic paper-based devices are manifold and compelling, particularly when benchmarked against conventional materials (Zhu et al., 2018). Paper as a substrate is not only economical and ubiquitously available but also lends itself to simple fabrication techniques conductive to mass production (Novo et al., 2014). These devices epitomize the pinnacle of portability and disposability, a boon for point-of-care diagnostics in remote settings where resources are scarce and logistical constraints are significant (Ozhikandathil et al., 2017). The analytical performance of these devices, particularly in terms of sensitivity and specificity, often matches or surpasses that of more traditional, resource-intensive platforms, as demonstrated by the development of a fully integrated rapid microfluidic device that translates conventional 96-well ELISA kits into point-of-care testing devices, significantly reducing reagent consumption and assay time while achieving higher sensitivity and specificity, (Uddin et al., 2021). The versatility of microfluidic paper-based devices is underscored by their compatibility with a diverse array of detection methods-from colorimetric to electrochemical- and their amenability to multiplexing, which enables simultaneous detection of multiple analytes (Jenkins et al., 2015; Tian et al., 2017).

Nevertheless, the path to widespread adoption and clinical integration of microfluidic paper-based devices is not without its challenges. Current limitations include variability in manufacturing quality, the necessity for precise fluid control, and the integration with electronic devices for data processing and readout (Rosenfeld and Bercovici, 2014). Furthermore, issues such as long-term stability of the reagents on the paper substrates and the need for enhancement of detection limits are pressing concerns that demand innovative solutions (Deng and Jiang, 2019).

Future research directions are both exciting and critical. They include exploring novel nanomaterials (Pereira et al., 2024) and bio-receptors like aptamers for improved sensitivity (Gandotra et al., 2023), developing advanced fluidic control mechanisms on paper (Sinha et al., 2022b), and integrating these devices with digital technologies for smarter diagnostics (Ahmed et al., 2016). The exploration of 3D paper-based devices to expand the range of possible assays and the push for further miniaturization and implementation of on-chip sample preparation steps to streamline workflows are also promising areas of development (Kuan et al., 2016).

Ultimately, this review aspires to provide a comprehensive understanding of the nuanced arena of microfluidic paper-based devices, their current capabilities, and future prospects in immunoassays. By distilling the essence of their benefits, tackling the technical bottlenecks head-on, and charting a course for future innovation, this analysis is poised to arm researchers and clinicians with the insights necessary to harness the full potential of these devices and navigate the evolving terrain of diagnostic technologies.

## 2 Materials and methods

**FIGURE 1:**
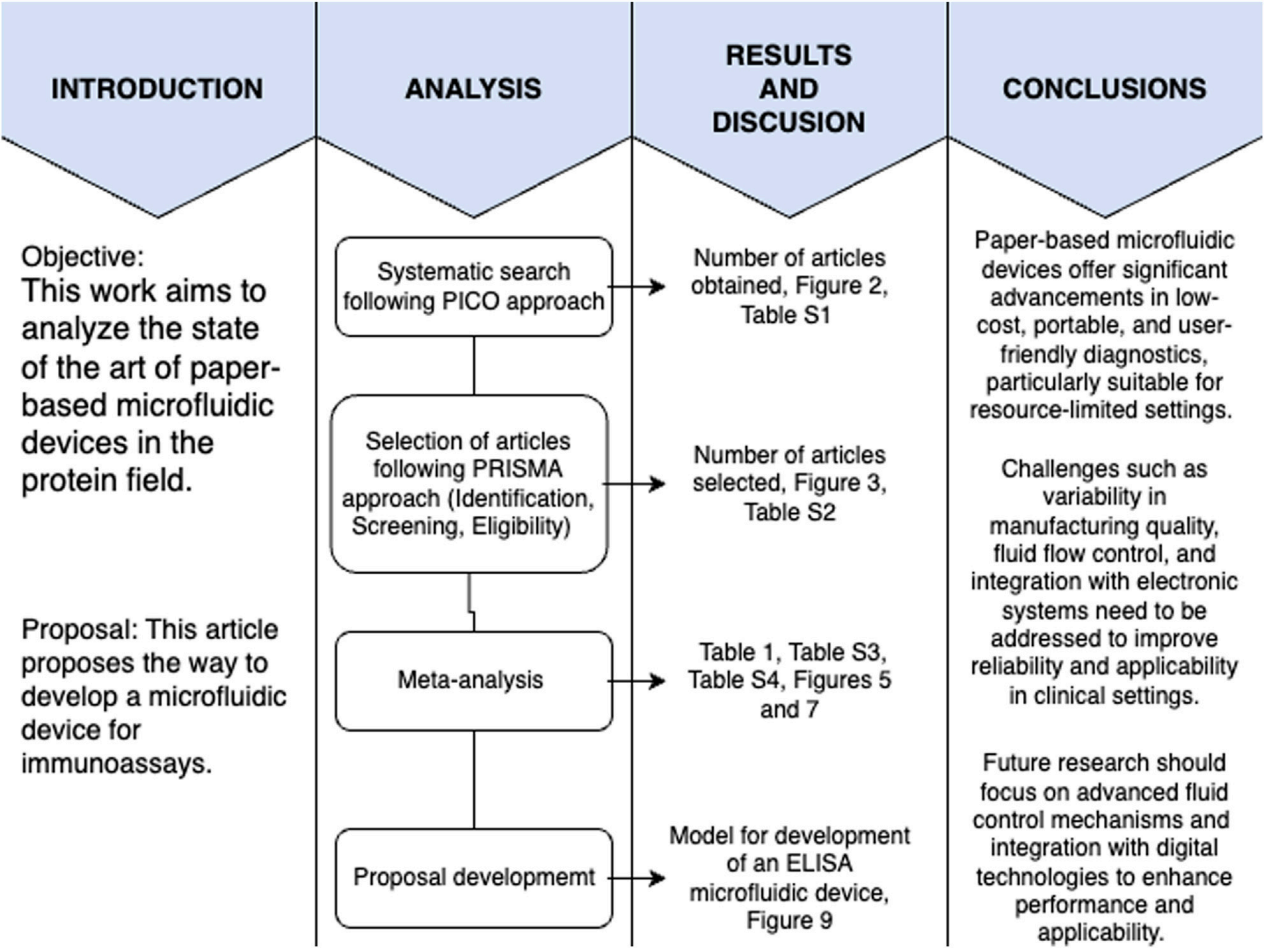
Methodology roadmap. The figure shows the steps taken to answer the research question.

A systematic review of all the scientific literature available until February 2024 was carried out for this study following the PICO (participants, intervention, comparison, and outcome) strategy and PRISMA (Preferred Reporting Items for Systematic Reviews and Meta-Analyses) approach (Liberati et al., 2009). A roadmap of the methodology followed in this study is shown in Figure 1, to guide the reader throughout the article and highlight which figures and tables belong to which sections. This figure depicts the objectives of the paper and the analysis followed to answer the research question, along with the results obtained and the pertaining discussion, followed by the conclusions made in the article.

### 2.1 PICO strategy

**FIGURE 2:**
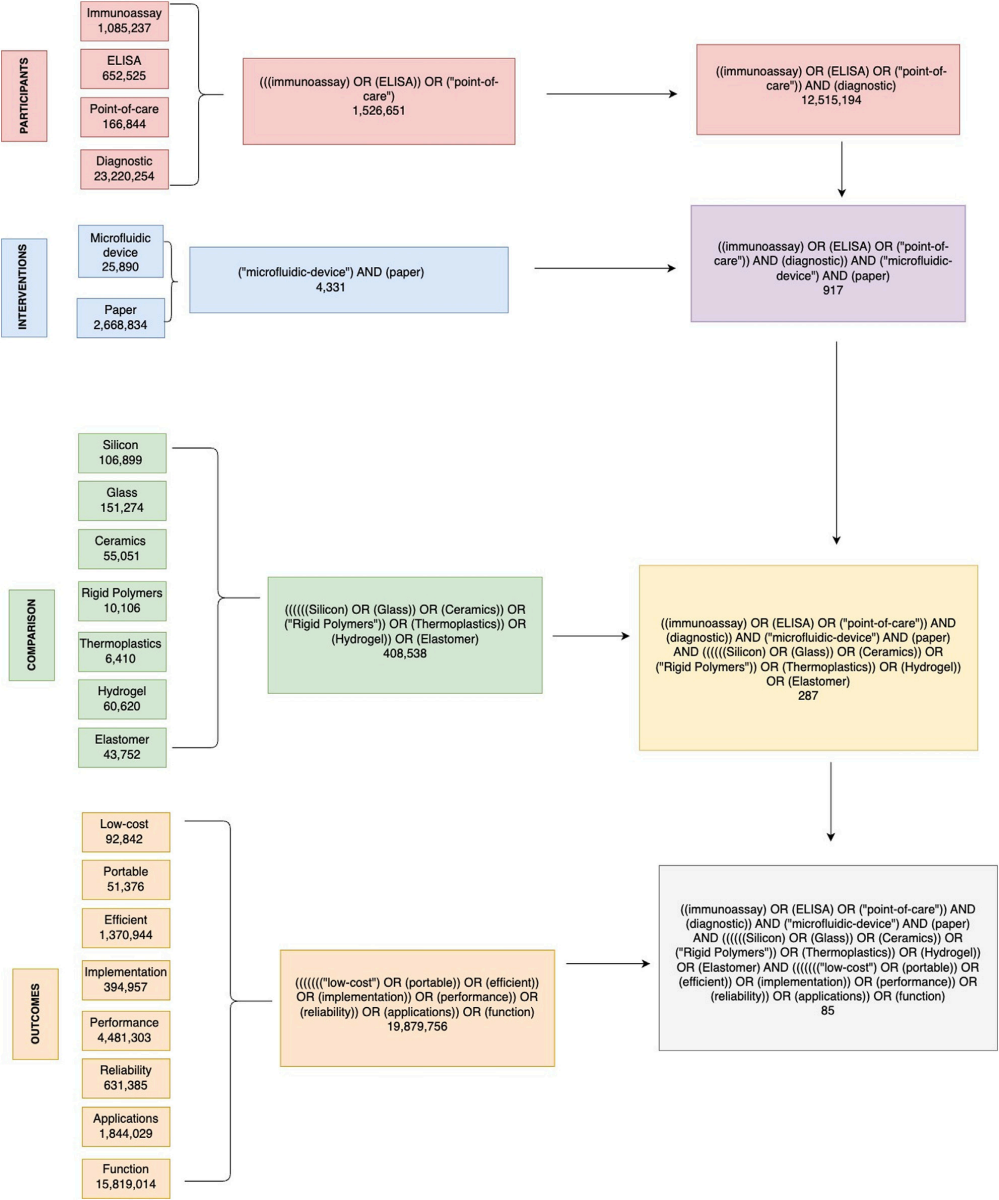
Decision tree diagram for identifying relevant literature on paper-based microfluidic devices in immunoassays. This figure illustrates the comprehensive search strategy used in this systematic review, based on the PICO (Participants, Interventions, Comparisons, Outcomes) framework. The flowchart outlines the search terms and combinations used to identify relevant articles from the PubMed database. P, participants; diagnostic, immunoassay, ELISA, Point-of-care. I, interventions: microfluidic device, paper. C, comparison: silicon, glass, ceramics, rigid polymers, thermoplastics, hydrogel, elastomer. O, outcome: Low-cost, portable, efficient, implementation, performance, reliability, applications, function.

The main objective is to analyse, explore and dive into the state of the art of microfluidic techniques and devices, in particular those that function as immunoassays. The PICO strategy (Figure 2.) was used to systematically search several databases and served as the methodology to answering the following research question: Among ELISA-based point-of-care immunoassay diagnostic tests (Participants), what is the current state of the art of microfluidic devices and paper-based platforms (Intervention), compared to various materials used for designing these devices (Comparison), in terms of design intricacies, operational efficacy, sensitivity, specificity, and challenges (Outcome)?

The public health in Mexico requires access to efficient, low-cost, reliable, and portable medical diagnostic methods. Small and marginalized regions do not have access to hospitals or clinical laboratories; therefore, the design and mass production of microfluidic based immunoassays represents an exciting opportunity to provide the necessary resources to these regions. The following terms were used using the PICO strategy to dissect the research question as participants, interventions, comparisons, and outcomes.

Participants: articles about immunoassays and their MeSH terms were considered for inclusion, with a particular emphasis on ELISA based diagnostics and point-of-care techniques:

• Immunoassay
• ELISA
• Point-of-care
• Diagnostic

Intervention: articles and studies that mentioned any type of microfluidic device and paper based microfluidic devices:

• Microfluidic device
• Paper

Comparison: articles that mentioned microfluidic devices based on the most commonly used materials other than paper:

• Silicon
• Glass
• Ceramics
• Rigid Polymers
• Thermoplastics
• Hydrogel
• Elastomer

Outcome: articles with the final desirable features for the microfluidic device:

• Low-cost
• Portable
• Efficient
• Implementation
• Performance
• Reliability
• Applications
• Function

### 2.2 Databases and searches

The electronic databases consulted were PubMed (free full-text archive managed by the National Institutes of Health’s National Library of Medicine), Web of Science (free full-text archive managed by Clarivate), and BIREME (specialized center managed by the World Health Organization and the Pan American Health Organization, whose Portuguese acronym stands for The Latin American and Caribbean Center on Health Sciences). The individual terms within each PICO category were searched using the Boolean operator “OR” except for “Diagnostic,” which was searched using the operator “AND” within the Participants category; each category was searched with the word “AND.” Specifically, the PICO strategy allows us to dissect the research question and its components. The Boolean operators “AND” and “OR” are used in a very specific manner: if we are interested in only what is similar to both sets, we use “AND” whereas if we are interested in either of the sets, we use “OR.” In this particular case, we used “AND” because we were only interested in diagnostics based on immunoassays. Furthermore, a decision tree was constructed to exemplify in full detail the search strategy used in the PubMed database, as well as the results obtained for each search algorithm with the number of resulting articles obtained. Subsequently, the results obtained were recorded and downloaded into Mendeley Reference Manager. This process was repeated exactly for the Web of Science and BIREME databases, after which all final articles were organized, eliminating duplicates for the final result.

### 2.3 Meta-analysis

This meta-analysis employs a qualitative synthesis approach to integrate epistemic and biochemical perspectives from the identified studies. Initially, a systematic review identified relevant literature based on epistemic and biochemical criteria. Selected studies were evaluated for their theoretical contributions and empirical support within both fields. The epistemic framework guided the qualitative synthesis, emphasizing conceptual coherence and theoretical insights drawn from biochemical data. Findings were synthesized narratively to explore the interplay between epistemic theories and biochemical evidence, focusing on theoretical implications and conceptual advancements rather than quantitative effect sizes. This approach aims to enrich understanding through a holistic integration of philosophical and empirical perspectives without relying on traditional statistical aggregation.

**FIGURE 3:**
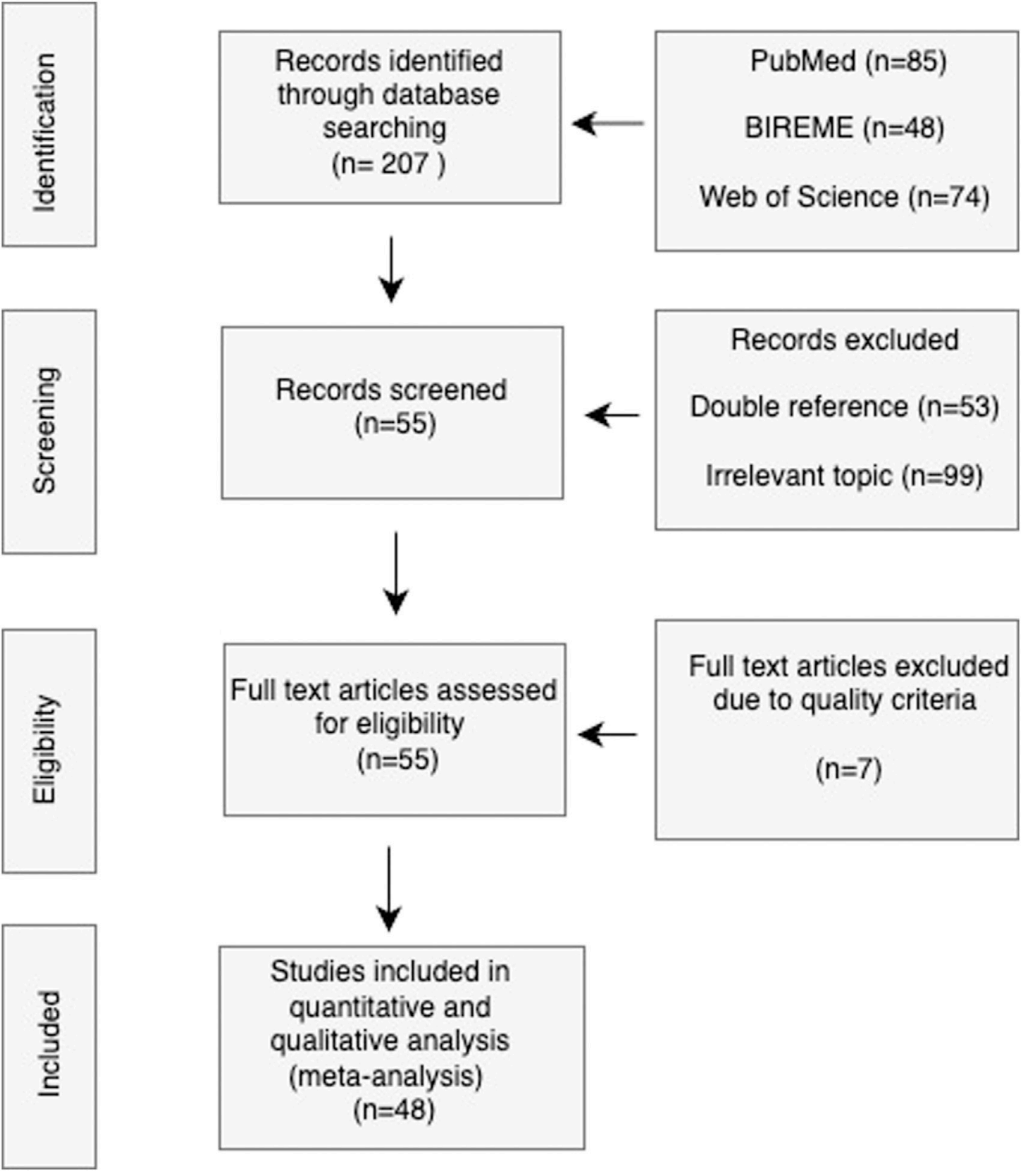
PRISMA flowchart for Study Selection Process. The diagram visually represents the meticulous process of narrowing down the initial broad search to a curated selection of high-quality studies that are relevant to the review’s objectives.

The meta-analysis was meticulously designed to synthesize data from a multitude of studies, thereby providing a quantifiable overview of the field. Initiating with a well-formulated research question, the process adhered to a structured flow chart as depicted in Figure 3. A total of 48 studies were scrupulously selected based on predetermined inclusion criteria such as relevance to immunoassays, use of paper-based microfluidic devices, and the sufficiency of data for meta-analysis. These studies were classified according to their distinct characteristics–namely, the type of assay conducted, the microfluidic device used, lithographic techniques employed, detection methods, sample mobility facilitators, and their application areas. In addition to these categories, studies were also evaluated on their comparison to benchtop systems and the rigor of their validation procedures, which include but are not limited to, sensitivity, specificity, and limit of detection metrics.

In the subsequent phase, data extraction focused on these classified variables, enabling a comparative analysis across the collected body of research. The quantification process involved tallying the occurrences of each category within the studies to identify prevalent trends, such as the most frequent utilized detection method or the commonality of a certain lithographic technique.

The meta-analysis culminated in a comprehensive narrative that distilled key findings form the quantitative data, providing actionable insights into current practices and pinpointing opportunities for innovation in the design and application of paper-based microfluidic devices in immunoassays.

## 3 Results: state of the art of paper-based microfluidic devices applied to immunoassays

### 3.1 Overview of selected studies

We present the results obtained from the systematic review, including the classification of the final 48 articles selected, their quality and finally the meta-analysis results.

207 records were obtained from the databases based on the search parameters used for the PICO strategy and PRISMA approach previously stated. Form this, 53 records were excluded due to them being doubly referenced and another 99 were excluded due to them being of irrelevant topic to the research question. We performed a PRISMA assessment of the quality of the included studies based on the following criterion:

a) Clear research objectives.
b) Research question in accordance with objectives.
c) Adequate methodology.
d) Definition of pertinent parameters.
e) Results according to objectives.

Each criterion was assigned a value of either 0 or 20% (See Table 1). Articles that presented a quality of 60% or below were not included for the meta-analysis. From 55 full text articles assessed for eligibility based on quality criteria seven articles were excluded due to their quality score, leaving 48 final studies included for the meta-analysis (see Table 1; Figure 3.), whose results are presented in the next section.

**TABLE 1:** Meta-analysis results.

Author, Year, Country	Assay type	Material	Detection method	Application	Validation
Pereira et al. (2024), Portugal	Drug Detection	Paper	Fluorescence	Healthcare Diagnostics	PCR/ELISA/CRISPR/IA/LAMP
Pan et al. (2024), Netherlands	Antigen Assay	Glass Fiber	Colorimetric Detection	Point-of-Care Testing	ELISA
Ko and Liao (2023), China	Fungi Assay	PDMS	Electrochemical	Clinical Diagnostics	ELISA/COVID-AG/LC-MS/SFM
Gandotra et al. (2023), Taiwan	Immunoassay	Silicon	ELISA	Pharmaceutical Analysis	A-BD/ELISA
Kumar et al. (2023), United States of America	Drug Detection	Cellulose	Optical-Color	DNA Sequencing	B-M/LOD/Sen/Spe
Sousa et al. (2023), Germany	Drug Detection	Paper	Fluorescence	Healthcare Diagnostics	B-M/CE
Heidari-Bafroui et al. (2023), United States of America	Nucleic Acid Detection	Hydrogel	SERS	Environmental Analysis	LOD/Sen/Spe
Jayachandran et al. (2024), United States of America	Antigen Assay	Glass Fiber	Colorimetric Detection	Point-of-Care Testing	ELISA
Toldrà et al. (2023), Sweden	Fungi Assay	PDMS	Electrochemical	Clinical Diagnostics	Sen/Spe/LOD/qRT-PCR/SeF/SF
Kumar et al. (2023), United States of America	Immunoassay	Silicon	ELISA	Pharmaceutical Analysis	LC-MS/ELISA
Mumtaz et al. (2023), Pakistan	Drug Detection	Cellulose	Optical-Color	DNA Sequencing	LOD/Sen/S-C/S-M
Pinheiro et al. (2023), Brazil	Drug Detection	Paper	Fluorescence	Healthcare Diagnostics	CE/MP/BioC/Sen/LOD/Spe
Li et al. (2023), China	Nucleic Acid Detection	Hydrogel	SERS	Environmental Analysis	SFM/LC-MS
Fu et al. (2023), United States of America	Antigen Assay	Glass Fiber	Colorimetric Detection	Point-of-Care Testing	SFM/BioC
Prat-Trunas et al. (2023), Spain	Fungi Assay	PDMS	Electrochemical	Clinical Diagnostics	ELISA/S-M
Wang et al. (2023), China	Immunoassay	Silicon	ELISA	Pharmaceutical Analysis	Sen/LOD/CE
Holman et al. (2023), Pakistan	Drug Detection	Cellulose	Optical-Color	DNA Sequencing	B-M
Wei et al. (2023), China	Drug Detection	Paper	Fluorescence	Healthcare Diagnostics	MP/Ac/BioC
Sinha et al. (2022b), India	Nucleic Acid Detection	Hydrogel	SERS	Environmental Analysis	B-M
Kim et al. (2023), Korea	Antigen Assay	Glass Fiber	Colorimetric Detection	Point-of-Care Testing	CE/B-M
Aralekallu et al. (2023), Korea	Fungi Assay	PDMS	Electrochemical	Clinical Diagnostics	B-M
von Stockert et al. (2021), Germany	Immunoassay	Silicon	ELISA	Pharmaceutical Analysis	B-M
Ni et al. (2021), China	Drug Detection	Cellulose	Optical-Color	DNA Sequencing	B-M
Li et al. (2020), China	Drug Detection	Paper	Fluorescence	Healthcare Diagnostics	CE/B-M
Ulep et al. (2020), United States of America	Nucleic Acid Detection	Hydrogel	SERS	Environmental Analysis	B-M
Theillet et al. (2019), France	Antigen Assay	Glass Fiber	Colorimetric Detection	Point-of-Care Testing	B-M
Deng and Jiang (2019), China	Fungi Assay	PDMS	Electrochemical	Clinical Diagnostics	B-M
Seth et al. (2018), Tanzania	Immunoassay	Silicon	ELISA	Pharmaceutical Analysis	CE/B-M
Kong et al. (2018), China	Drug Detection	Cellulose	Optical-Color	DNA Sequencing	B-M
Tian et al. (2017), China	Drug Detection	Paper	Fluorescence	Healthcare Diagnostics	CE/B-M
Hegener et al. (2017), United States of America	Nucleic Acid Detection	Hydrogel	SERS	Environmental Analysis	CE/B-M
Ozhikandathil et al. (2017), Canada	Fungi Assay	PDMS	Electrochemical	Clinical Diagnostics	B-M
Kuan et al. (2016), China	Immunoassay	Silicon	ELISA	Pharmaceutical Analysis	CE/B-M
Shatova et al. (2016), United States of America	Drug Detection	Cellulose	Optical-Color	DNA Sequencing	CE/B-M
Wei et al. (2016), China	Drug Detection	Paper	Fluorescence	Healthcare Diagnostics	B-M
Malekghasemi et al. (2016), Turkey	Nucleic Acid Detection	Hydrogel	SERS	Environmental Analysis	B-M
Wei et al. (2015), China	Antigen Assay	Glass Fiber	Colorimetric Detection	Point-of-Care Testing	CE
Niedl and Beta (2015), Germany	Fungi Assay	PDMS	Electrochemical	Clinical Diagnostics	CE
Rosenfeld and Bercovici (2014), Israel	Immunoassay	Silicon	ELISA	Pharmaceutical Analysis	B-M
Novo et al. (2014), Portugal	Drug Detection	Cellulose	Optical-Color	DNA Sequencing	B-M
Park et al. (2013), United States of America	Drug Detection	Paper	Fluorescence	Healthcare Diagnostics	B-M
Lin et al. (2011), China	Nucleic Acid Detection	Hydrogel	SERS	Environmental Analysis	CE/B-M
Theberge et al. (2010), Germany	Antigen Assay	Glass Fiber	Colorimetric Detection	Point-of-Care Testing	CE/B-M
Huebner et al. (2009), United Kingdom	Fungi Assay	PDMS	Electrochemical	Clinical Diagnostics	CE/B-M
Bhattacharyya and Klapperich (2007), United States of America	Immunoassay	Silicon	ELISA	Pharmaceutical Analysis	B-M

Supplementary Table S1 presents a comprehensive quality assessment of the 55 articles selected for inclusion in the meta-analysis. It highlights the preeminence of China and the United States of America in the field, contributing 27% and 20% of the pivotal research, respectively. The literature predominantly features immunoassays, drug detection, and antibody-based assays, signifying the vital role these applications play in the advancement of paper-based microfluidic devices.

Immunoassays, constituting 15% of the studies, utilize microPADs for the detection of antigens or antibodies, demonstrating their utility in diagnosing infectious diseases, managing chronic conditions, and even orchestrating population-wide screening programs. Their high sensitivity and specificity make them invaluable for early disease detection and monitoring therapeutic responses.

Drug detection, another prominent application found in 13% of the studies, underscores the capacity of microPADs to identify and quantify pharmaceuticals and illicit substances. This capability is critical in fields ranging from clinical pharmacology, where therapeutic drug monitoring is essential, to law enforcement and public health, where rapid on-site testing can have profound implications.

Antibody-based assays, equally accounting for 13%, are pivotal in identifying specific proteins or biomarkers, providing to be instrumental in applications such as cancer diagnostics, autoimmune disease identification, and allergen detection. The convenience and speed of these assays on paper-based devices facilitate point-of-care diagnostics and enable continuous patient monitoring without the need for sophisticated lab equipment.

The table also cites a diverse range of other applications, with point-of-care testing leading at 20%. These devices enable critical diagnostics in resource-constrained environments, supporting efforts in global health initiatives by providing diagnostic tools that are both accessible and reliable. Clinical diagnostics, forming 12% of the applications, benefit from the quick turnover and ease of use of microPADs, accelerating patient care through faster diagnostics and enabling the monitoring of disease progression in real-time.

In summary, the versatility and accessibility offered by paper-based microfluidic devices are game-changers across diverse scientific and medical fields. Each application demonstrates how microPADs are not only reshaping diagnostics and therapeutics but also pushing the boundaries of what is possible within and beyond the laboratory.

Supplementary Table S2 lays out a detailed synopsis of the final 48 articles included in the meta-analysis, showcasing a landscape where paper-based microfluidic devices take center stage in experimental design. These devices are ingeniously adapted for a multitude of applications, with some studies integrating hydrogels into the device architecture. Hydrogels serve multifarious functions: as reservoirs for analytes, enhancing the bioactivity and shelf life of the reagents (von Stockert et al., 2021), modulating the fluidic flow to ensure precision in the delivery and processing of the sample (Wei et al., 2015; Gandotra et al., 2023), and expanding the capacity of detection zones, which is critical for high-throughput screening applications (Niedl and Beta, 2015). This adaptability is augmented by the affordability of paper as a substrate, with (Yetisen et al., 2013) providing a cost-effective benchmark for paper-based assays which, despite regional cost variances, highlights the economic efficiency of this medium.

Further enhancing the applicability of these devices, the diversity in detection methods unveiled by the reviewed studies underscores the technological breadth of µPADs. Optical detection remains the most prevalent, owing to its straightforward implementation and the direct relationship between color change or fluid progression and analyte concentration. Such a direct readout offers immediate visual feedback, which is especially beneficial in settings with limited access to sophisticated equipment. Beyond colorimetric and distance-based methods, other innovative optical techniques, such as fluorescence and chemiluminescence, have been harnessed to achieve even greater sensitivity and specificity.

The validation of these devices is as varied as their design, with benchmarks set against traditional assays to determine their diagnostic accuracy. The reviewed articles detail comparative studies, where µPADs are pitted against gold-standard laboratory techniques to evaluate performance metrics such as limit of detection (LOD), specificity, and sensitivity. These studies often reveal that µPADs not only match but sometimes exceed the capabilities of conventional assays, all while offering the additional benefits of portability and cost-effectiveness. For instance, (Fu et al., 2023; Heidari-Bafroui et al., 2023), have demonstrated that the LOD in paper-based devices can reach the nano- or even picomolar range, rivalling the sensitivity of more complex lab-based systems.

In addition, the utility of µPADs in point-of-care settings is consistently emphasized, where rapid, reliable, and reproducible diagnostics are paramount. The simplicity and rapidity of the detection methods embodied in µPADs enable their deployment in the field, delivering critical diagnostic information in real-time, thus facilitating immediate clinical decision-making. This translates into a powerful impact on public health, particularly in under-resourced areas or in scenarios where prompt responses to emergent health threats are required.

### 3.2 Classification of paper-based microfluidic devices

Workflow Procedure.

**FIGURE 4:**
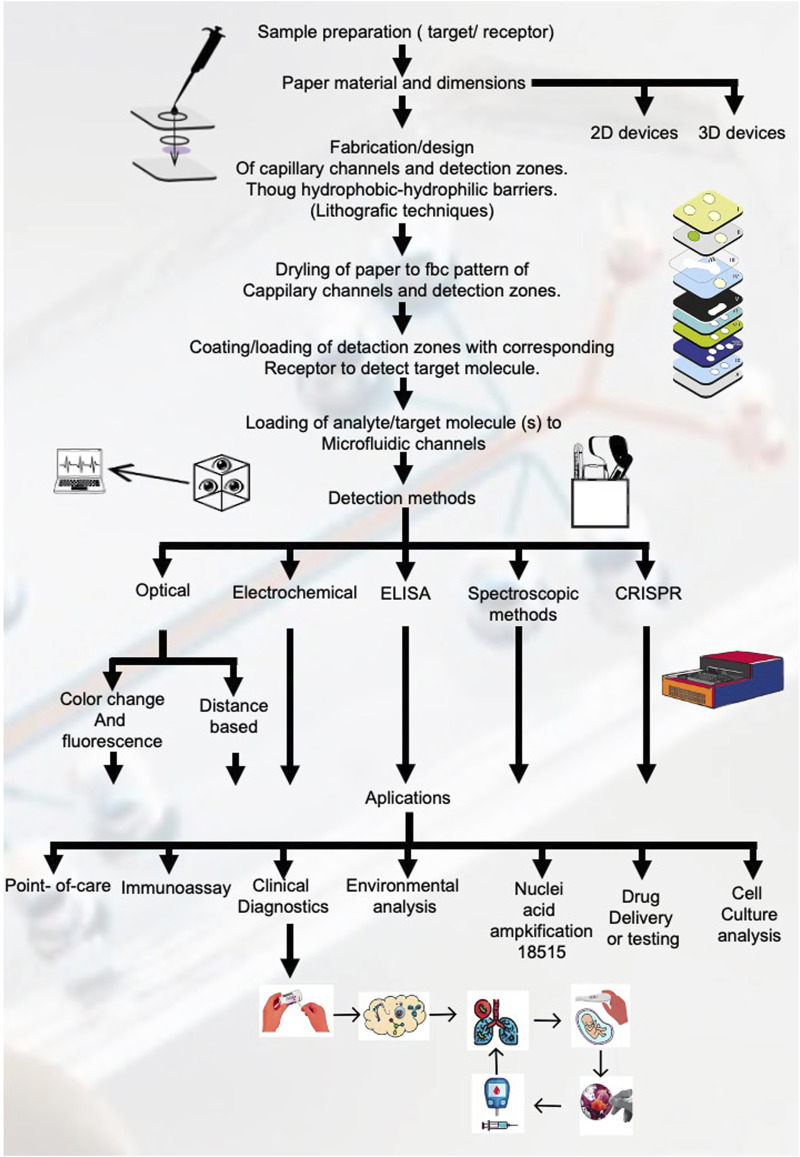
Workflow for fabrication and application of paper-based microfluidic devices. This figure outlines the comprehensive workflow for the fabrication and application of paper-based microfluidic devices, showing each critical step from sample preparation to detection methods and applications.

In Figure 4 all the pathways for the fabrication and design of paper-based microfluidic devices.

1) Selection of the microfluidic device material, in the case of paper-based microfluidic devices, the most used materials found from the systematic review results are Whatman No.1 filter paper, cellulose chromatography paper and nitrocellulose membranes.
  a) The next step is to decide the device dimensions and type, that is, 2D device or 3D device. In a 2D device, the fluid flows via capillary action horizontally though the microfluidic channels, whereas in a 3D device, the fluid travels both horizontally and vertically through the different layers of paper material stacked on top of each other (Tian et al., 2017; Wei et al., 2023).
2) Design of micro-scale microfluidic channels and detection zones through hydrophobic-hydrophilic barriers. This step can be accomplished through various techniques such as wax printing, photolithography, inkjet printing, laser cutting, etc.
3) Drying of paper using a conventional hot plate or oven to fix the pattern of the microfluidic channels and detection zones.
4) Coating/loading of detection zones with corresponding receptor to detect target molecule.
5) Loading of analyte/target molecule(s) to microfluidic channels. The main driving force for this step is the mobility of the sample by capillary action through the channels, but other methods can be employed to enhance or control the mobility such as the use of syringe pumps (Huebner et al., 2009; Shatova et al., 2016), micro pumps (Ozhikandathil et al., 2017), electro-osmotic flow (Aralekallu et al., 2023) and sequential (Novo et al., 2014) or segmented flow techniques (Theberge et al., 2010).
6) Critical step. The detection and quantification of the analyte can be achieved through various techniques, the most common being optical detection, whether it be colorimetry based, distance based or a combination of both. One of the advantages of this method relies on its relative simplicity, this being that the intensity of colour change is directly proportional to the analyte concentration. Other methods commonly used are fluorescence based, electrochemical methods, ELISA, digital image analysis using a digital camera or smartphone, etc. Most of these methods are adequate for point-of-care settings due to their simplicity, handling, cost, and portability. Other more complex methods are detailed in Table 2.
7) A great number of applications exist for µPADs such as point-of-care diagnostics, immunoassays, environmental analysis, nucleic acid amplification tests, drug testing, cell culture, etc. and even though the design and implementation of each µPAD is specific to the intended assay and application beforehand, we find that a key feature of these systems is the adaptability to perform multiple tests in the same platform; the only variation being the specific reactants, analytes and target molecules for each assay conducted.

**TABLE 2:** An overview of microfluidic devices: database search using filter: in the last year (2023–2024): features.

Author	Microfluidic platform	Improvement	Processing time	Characteristics
Pereira et al. (2024)	MicroPADs	Incorporation of silica nanoparticles to enhance color uniformity and intensity of the detection reaction	25 min	Excellent repeatability, user-friendly, low-cost
Lee et al. (2023)	Lateral flow assay strips, microfluidic channels, paper-based microfluidic devices	Integration of microfluidic devices with portable optical readers	NA	High sensitivity and specificity, rapid results
Pan et al. (2024)	MicroPADS	Use of black phosphorus-Au (BP-Au) nanocomposites to enhance the electron transfer rate thereby amplifying the detection signal	20 min	Reproducibility, sensitivity and specificity
Ko and Liao (2023)	MicroPADs Digital droplet	High Integration High Accuracy	<60 min <15 min	Paper-based sugar valve *In situ* array heater
Gandotra et al. (2023)	Paper-based aptamer-sandwich assay	Reduction of the assay operation time	42 min	High sensitivity, streamlined process
Kumar et al. (2023)	Sample in-result-out integrated microfluidic platform Multiplexing platform	High Integration	<90 min	Pressure driven, parallel channel multiplexing
Sousa et al. (2023)	MicroPADs	Dual mode signal readout sensing strategy	<60 min	Use a saliva sample directly
Heidari-Bafroui et al. (2023)	Lab-on-paper device for performing ELISA	Integration of B-MaC, based on the mechanics of a two-material cantilever bean	40 min	Sensitivity and Specificity, reproductibility
Jayachandran et al. (2024)	Direct flow control platform	High sensitivity and accuracy	20 min	Forward type of blood grouping
Toldrà et al. (2023)	MicroEL-PAD	Universal paper-based technology suitable for high-sensitivity quantification of various analytes	NA	Versatility, quantitative analysis
Kumar et al. (2023)	Micro-PADs	Paper-based bi-material cantilever	NA	Response to fluid imbibition, low-cost, biodegradable
Mumtaz et al. (2023)	Microfluidic platforms designed for nucleic acid detection	Isothermal amplification combined with lateral flow assays	NA	Cost-effectiveness, ease of use
Pinheiro et al. (2023)	Microfluidic system designed for the detection of neglected topical diseases	Inexpensive materials such as paper and polyester	NA	Integration of functions, versatility
Li et al. (2023)	MicroPADs	High integration	<60 min	Paper based, sugar valve
Fu et al. (2023)	Electrochemical microPADs	High sensitivity	NR	All-in-one origami design
Prat-Trunas et al. (2023)	Electric chemistry microfluidic	High sensitivity	20 min	Graphene and poly-lysine materials
Li et al. (2023)	Centrifugal microfluidic platform	High accuracy and integration	<60 min	Capillary action driven, multifunctional agarose bead strategy
Holman et al. (2023)	MicroPED	Integration of diverse materials and methods for fabricating hydrophobic barriers and electrodes	NA	Ease of fabrication, low cost and portability

The results obtained from the meta-analysis exemplify the growth that has occurred in recent decades in terms of applicability for microfluidic systems. Tables 1, 2 and Figure 4 show the different assays, materials used for the microfluidic devices, lithographic techniques and applications found in the 48 articles included in the review. A total of 20 different assays were performed, with the most common one being immunoassays, accounting for 15% of the total count or assays mentioned in the articles. In terms of the materials used, 49% are paper based from a total of 31 different materials found in the articles. Using paper as the microfluidic devices’ substrate has gained interest due to their low-cost, ease of handling, specificity, sensitivity, biocompatibility, etc. all of which are essential criteria when designing a microfluidic device for point-of-care settings.

The specificity of a microfluidic system is determined by its ability to detect and differentiate positive signals produced from the analyte-receptor reaction from the negative signals that arise form the noise of the system, which most commonly occur due to the sample fluid containing multiple molecules and the receptor not having a high specificity to the analyte or target molecule. Reducing the noise leads to more reliable results, so it is of utmost importance to consider this when designing and implementing a microfluidic assay. Sensitivity here refers to the lowest concentration of analyte detectable during the microfluidic assay, also known as limit of detection (LOD). One of the main advantages of microfluidic assays is the handling of small sample volumes, therefore reducing the LOD attained from the system allows for better assays utilizing fewer sample volumes and reducing the overall cost. Biocompatibility is another trait of these systems that has great importance when considering their performance and it is crucial for assays that utilize biological samples as the analyte and/or receptor, such as immunoassays, DNA sequencing, cell culture, etc. This means that for the microfluidic device to be biocompatible, there can be no unwanted or adverse effects to the biological sample introduced to the system, meaning that the substrate material of the device should not react with the sample or target molecule (Wei et al., 2023). designed a biocompatible Paraflim®-based 3D device. To validate the materials biocompatibility they cultivated 100 μL of *E. Coli* bacteria suspension for 7 days inside the microfluidic chip or device, obtaining results similar to bacterial cultivation in typical liquid culture.

#### 3.2.1 Aptamers as an alternative to antibodies in immunoassays

Aptamers are emerging as a viable alternative to antibodies in immunoassay applications, offering several advantages that are highlighted across various studies (Gandotra et al., 2023). Unlike antibodies, aptamers are synthetic oligonucleotide sequences that can fold into unique three-dimensional structures, enabling specific binding to target molecules, including proteins and small molecules (Pan et al., 2024). Consequently, the integration of aptamers into microfluidic devices, as exemplified by the development of a microfluidic paper-based analysis device for the selective detection of peanut allergen Ara h1, underscores the potential of aptamers to revolutionize diagnostic assays by combining the specificity and sensitivity of traditional immunoassays with the versatility of synthetic biorecognition elements.

#### 3.2.2 Including nanoparticles and nanocomposites in microfluidic devices for signal amplification

The integration of nanoparticles and nanocomposites into paper-based microfluidic devices presents a transformative approach to enhancing the analytical performance of these platforms. The employment of black phosphorus-Au nanocomposites, for instance, markedly improves the electron transfer rate at the electrode interface, thereby amplifying the signal detection for target analytes such as the peanut allergen Ara h1. This innovative application not only contributes to achieving a lower detection limit but also extends the linear response range, facilitating more sensitive and accurate assays (Pan et al., 2024). The inherit properties of nanoparticles, including their high surface area-to-volume ratio and the ability to modify their surface with various functional groups, enable a more efficient immobilization of biomolecules, such as aptamers, thereby enhancing the specificity and stability of the detection method. Additionally, the use of nanocomposites and nanoparticles allows for the incorporation of various functionalities within a single platform, integrating signal amplification and target recognition elements, which significantly improves the overall performance of paper-based microfluidic assays (Pereira et al., 2024).

### 3.3 Meta-analysis

**FIGURE 5:**
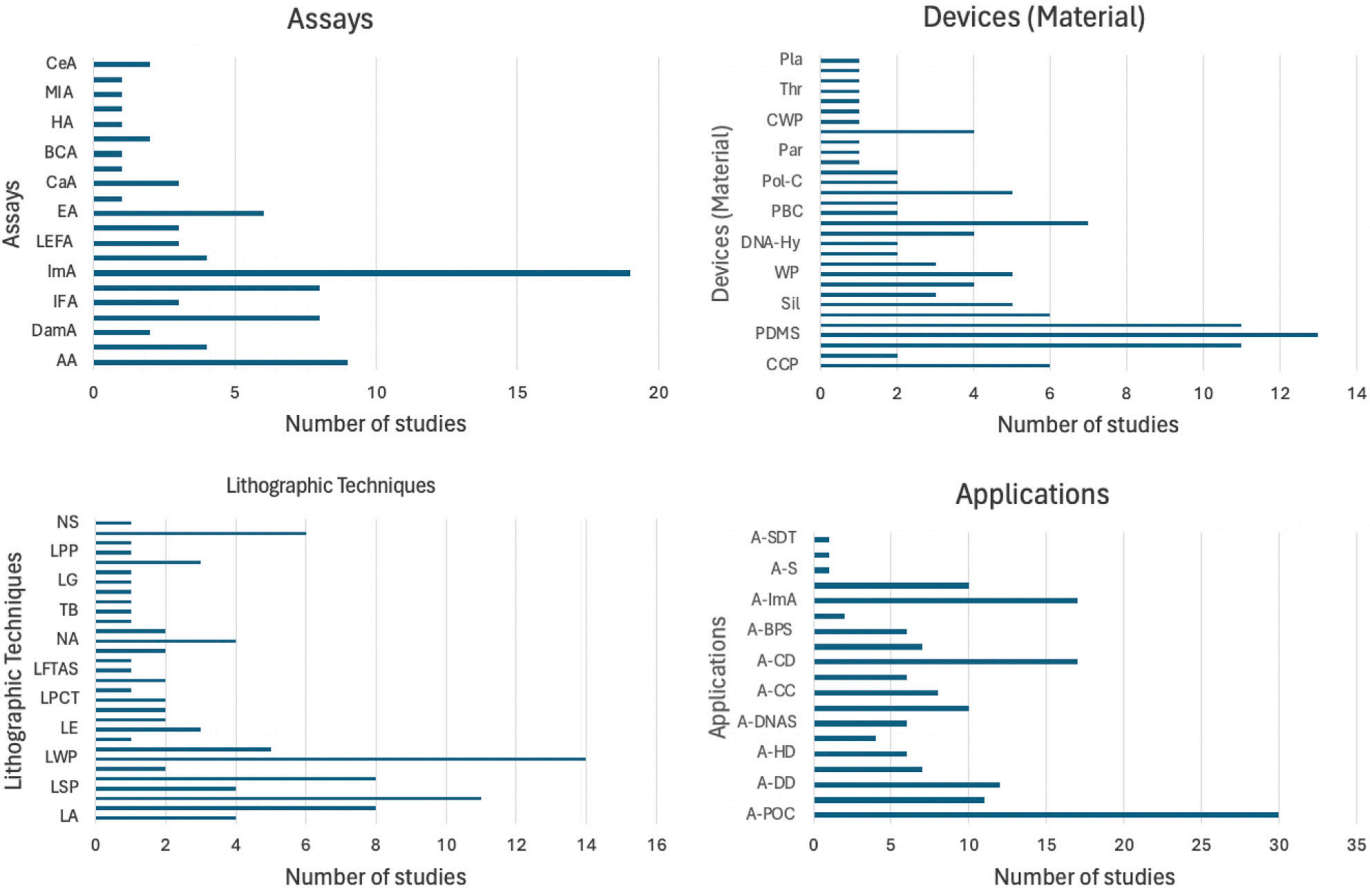
Distribution of studies based on assay types, device materials, lithographic techniques, and applications. The bar graphs summarize the methodological diversity and application breadth of microfluidic devices, demonstrating their versatility.

Figure 5 provides a comprehensive overview of the methodological diversity and wide-ranging applications of microfluidic devices. The bar graphs summarize findings from Table 1, which compiles results from a meta-analysis of recent studies such as those by Kim et al. (2023); Pereira et al. (2024), and others. These studies encompass a global scope, from Portugal to China, examining a variety of assay types including immunoassays (ImA), nucleic acid detection (NAD), and antigen lateral flow assays (AgLFA). Device materials range from polydimethylsiloxane (PDMS) to cellulose chromatography paper (CCP), utilized in conjunction with advanced lithographic techniques like photolithography (PL) and soft lithography (SL). Applications of these technologies extend to point-of-care diagnostics (A-POC), cancer detection (A-CaD), and drug delivery/testing (A-DD), demonstrating the versatility and impact of microfluidics in modern biomedical research and clinical practice.

#### 3.3.1 Scope of fabrication techniques

##### 3.3.1.1 Wax printing

**FIGURE 6:**
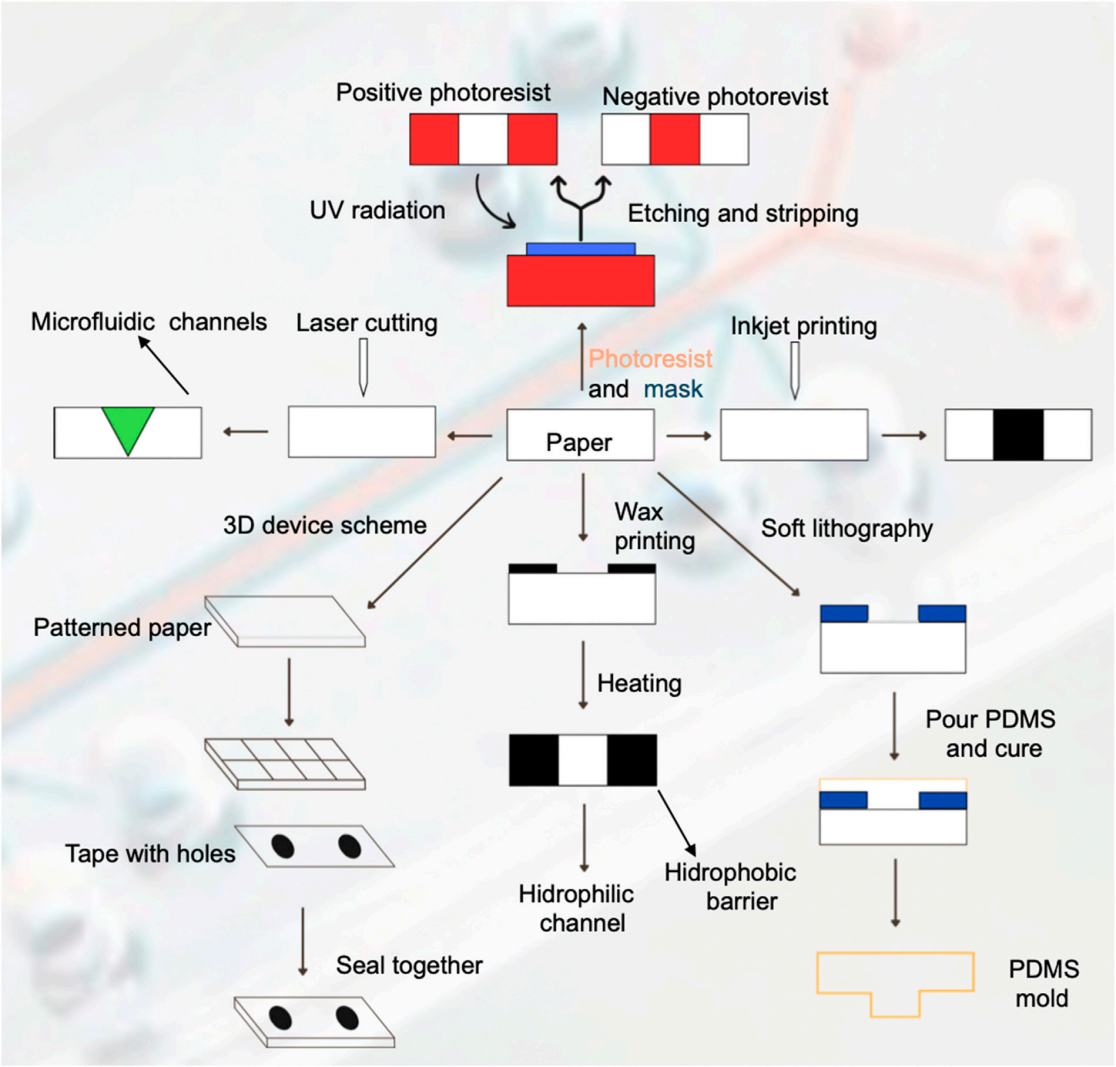
Fabrication techniques for paper-based microfluidic devices. This figure illustrates various fabrication techniques used to create paper-based microfluidic device, depicting the steps involved in each method and the resulting structures.

Wax printing presents advantages such as a simple and fast fabrication process, utilizing solid wax as the raw material and a wax printer as well as a hot plate or conventional oven to melt the wax, which in turn creates a hydrophobic barrier to allow the fluid to flow though the capillary or hydrophilic channels. This technique has certain disadvantages such as poor availability in resources, expensive material and low-resolution during detection (Sinha et al., 2022a). Several authors (Rosenfeld and Bercovici, 2014; Wei et al., 2015; 2016; Tian et al., 2017; Deng and Jiang, 2019; Ulep et al., 2020; Ni et al., 2021; Fu et al., 2023; Sousa et al., 2023; Wang et al., 2023) utilized this technique to fabricate their microfluidic devices (Figure 6).

##### 3.3.1.2 Photolithography

Photolithography is one of the most used fabrication techniques due to its high resolution of microfluidic channels, however, it requires expensive reagents and equipment, as well as complex steps for the fabrication procedure. It utilizes a photoresist solution and ultraviolet resin as the raw material and lithography equipment, a UV light source and hot plate or conventional oven (Sinha et al., 2022a). The process utilizes UV irradiation to expose the pattern of microfluidic channels into the photoresist coating and transfers it to the paper substrate. The removal of the exposed photoresist with a solvent corresponds to a positive photoresist, and the removal of the unexposed photoresist corresponds to a negative photoresist. Finally, the use of a hot plate or conventional oven hardens the photoresist layer to produce the microfluidic channels. Several authors (Lin et al., 2011; Novo et al., 2014; Shatova et al., 2016; Tian et al., 2016; Lepowsky et al., 2017; Iwai et al., 2022; Sinha et al., 2022b; Mumtaz et al., 2023) utilize or review this fabrication technique.

##### 3.3.1.3 Inkjet printing

This technique firstly requires a hydrophobic solution to saturate or coat the paper-based microfluidic device. Afterwards, a modified inkjet printer is utilized to imprint a hydrophilic solution onto the substrate to generate the microfluidic channels. It provides high resolution of the desired pattern, so it is a very attractive fabrication technique. Several authors (Malekghasemi et al., 2016; Lepowsky et al., 2017; Deng and Jiang, 2019; Sinha et al., 2022b; Prat-Trunas et al., 2023) utilize or review this fabrication technique.

##### 3.3.1.4 Laser cutting

This fabrication technique yields sharp defined features for the microfluidic channels onto the paper substrate, but it requires expensive equipment and can produce low mechanical stability. The most used equipment is a computer-controlled CO_2_ laser cutter. Theillet et al. (2019) used this technique to fabricate their glass-fiber laser cut microfluidic device to detect chikungunya virus-specific IgM, obtaining sensitivity results of 70.6% and a specificity around 98%.

##### 3.3.1.5 Soft lithography

Soft lithography utilizes an elastomer such as PDMS as a stamping agent on the microfluidic device surface to produce de desired microfluidic pattern or channels and it can also produce a soft PDMS mold. The advantage of this technique is that the patterning agent is cheap, but there is less control in terms of the hydrophobic barrier properties (Novo et al., 2014; Sinha et al., 2022a) used this technique to fabricate a microfluidic device that integrates photodiodes to perform a model chemiluminescence ELISA assay, obtaining a limit-of-detection of around 2 nM. Courtois et al. (2008) also utilized soft lithography to fabricate their microfluidic device for the trapping, incubation and release of droplets for enzymatic and cell-based assays. They obtained single cell level limit of detection.

##### 3.3.1.6 3D devices

This technique generally consists of stacking of multiple patterned pads or layers on top of each other or 3D wax printing, which extends the traditional wax printing technique described before to produce multiple layers to form three-dimensional microfluidic channels (Sinha et al., 2022b; Kumar et al., 2023).

**FIGURE 7:**
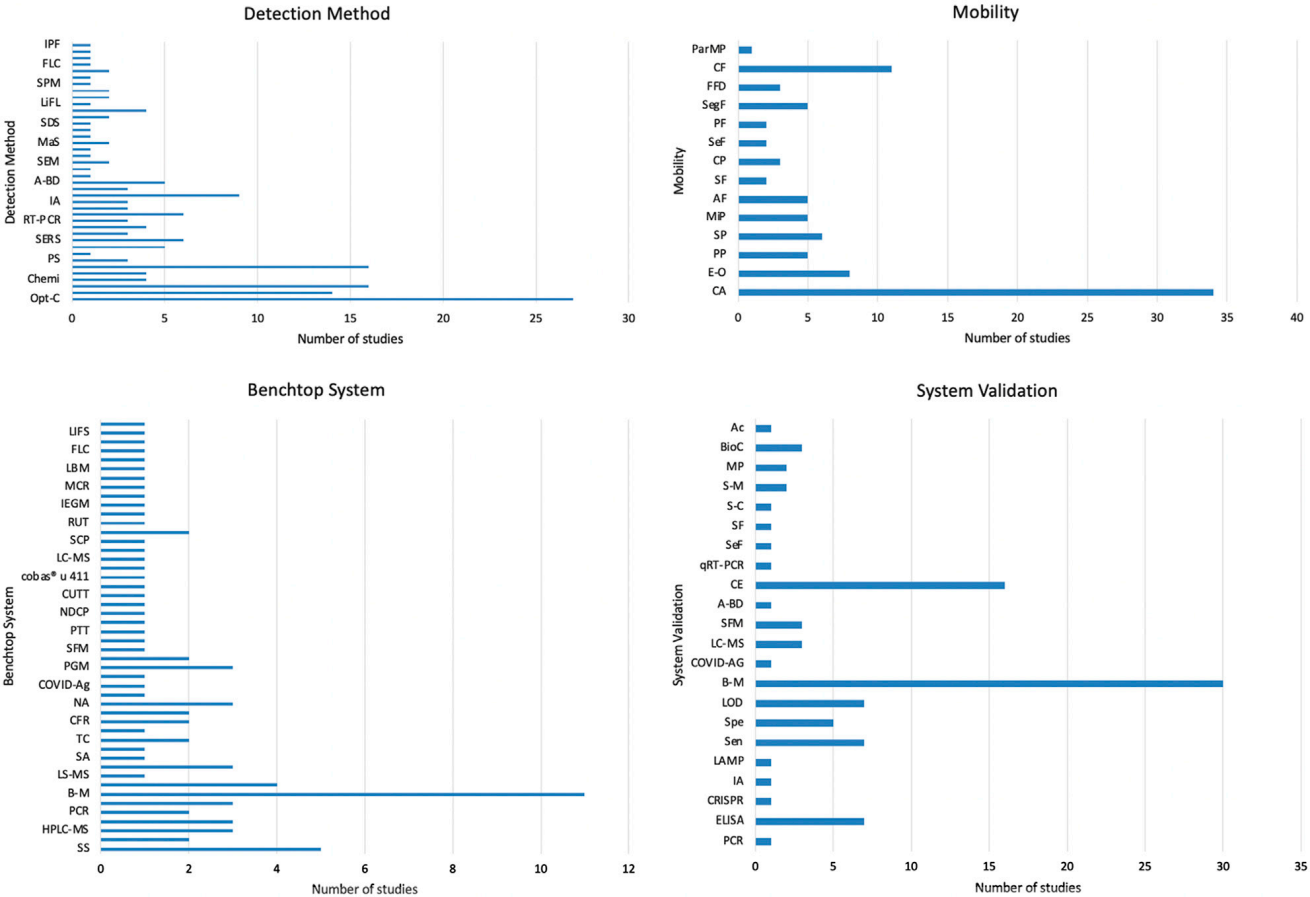
Analysis of detection methods, mobility, benchtop systems, and system validation in microfluidic devices. These bar graphs provide a comprehensive overview of the technological and methodological diversity in the field of microfluidic devices, emphasizing the different approaches and standards to develop these innovative diagnostic tools.

Supplementary Table S3 and Figure 7 present the rest of the meta-analysis results, showing the different detection methods, type of fluid mobility, benchtop systems, microfluidic system validation and the sensitivity and detection limits. As stated before, optical-based detection methods represent the most used technique, accounting for 18% (optical-color), 9% (fluorescence) and 8% (optical-distance) of the total amount of detection methods found in the review. In terms of fluid mobility, 67% of the microfluidic devices rely on capillary action or force for the fluid sample to flow through the microfluidic capillary channels, therefore avoiding the need to use micropumps or microvalves that serve the same purpose. The benchtop systems used to validate the assay results of the microfluidic devices varied a lot, with the most common one being a commercially available personal glucose metre (PGM), accounting for 7% of the total benchtop systems found in the literature reviewed (Tian et al., 2017; Deng and Jiang, 2019; Gao et al., 2020). The increased performance of these systems can be exemplified through the results obtained from the sensitivity and detection limits, where most results range in the micro-scale. Ulep et al. (2020) obtained a limit of detection of 0.1 cells/μL utilizing smartphone fluorescence imaging of cancer cells in their dual-layer paper microfluidic chip integrating smartphone imaging. The lowest LOD found in the articles reviewed is reported by Kong et al. (2018), achieving a sensitivity in the part-per-billion scale, specifically 1 ppb of pyrene from mixed sample with Raman dye and 10 ppb of cocaine from human plasma. The unprecedented sensitivity was achieved through the implementation of microfluidic diatomite analytical devices (μDADs), being able to simultaneously perform on-chip chromatography to separate smaller molecules form the complex biofluidic sample and perform the surface-enhanced Raman scattering spectra of the target molecules to achieve that LOD. As the development of microfluidic devices continues, lower LODs will and higher specificities will result in almost ideal point-of-care testing platforms, paving the way for accessible and low-cost diagnostics for small and marginalized regions.

### 3.4 Latest advancements in microfluidic devices

Table 2 presents an overview of the features of various microfluidic devices from multiple studies from the last year. Pereira et al. (2024) have developed MicroPADs that incorporate silica nanoparticles to enhance color uniformity and intensity, taking 25 min for the process with characteristics like excellent repeatability, user-friendliness, and low-cost. Pan et al. (2024) also worked MicroPADs, using black phosphorus-Au nanocomposites to amplify detection signals, achieving the process in 20 min with noted reproducibility, sensitivity, and specificity. Gandotra et al. (2023) created a paper-based aptamer-sandwich assay that reduced the assay operation time to 42 min, featuring high sensitivity and a streamlined process. Sousa et al. (2023) developed MicroPADs with a dual-mode signal readout sensing strategy, operable in under 60 min and capable of directly using saliva samples. Jayachandran et al. (2024); Toldrà et al. (2023) focused on enhancing sensitivity and offering versatility for quantitative analysis in their respective microfluidic platforms. Not all entries provided process times or described the characteristics in detail, and for some, such as Holman et al. (2023), the emphasis was on the ease of fabrication and the integration of diverse materials for constructing hydrophobic barriers and electrodes.

The advancements in electrochemical paper-based microfluidic devices (μPEDs) are illustrated through a detailed diagram that showcases their innovative features. The diagram includes several key components and processes: (a) the Microfluidic Device, which highlights the intricate design and components of a paper-based electroanalytical device; (b) How the Immunoassay Works, depicting the step-by-step operational process for electrochemical measurement of alpha-fetoprotein using a paper-based device; (c) Fluid Manipulation, which demonstrates the role of hydrophobic barriers in enabling precise fluid control on a single plane; (d) Detection and Results, providing an illustration of the process for detecting avian influenza with a microPED; and (e) Potential Applications, focusing on environmental applications such as a vertical-flow paper electrochemical sensor for monitoring chloride contamination. These μPEDs leverage the unique properties of paper, such as capillary action for fluid transport, hydrophobic patterning for channel formation, and integration of electrochemical sensors, to create low-cost, disposable, and highly efficient devices suitable for point-of-care diagnostics and environmental monitoring. The article by Holman et al. (2023) delves into the various fabrication techniques, functional components, and diverse applications of these devices, underscoring their potential to revolutionize the field of electrochemical sensing in paper-based electrochemical devices (Holman et al., 2023).

The latest advancements in microfluidic technology are exemplified in the diagram, highlighting the sophisticated design and functional capabilities of microfluidic chips for biomedical applications. (a) Microfluidic Device: The image depicts a microfluidic chip capable of accommodating seven samples, each within its own channel, featuring molded holes for the attachment of reagent-loaded tubes. (b) How the Immunoassay Works: This illustration outlines the biochemical reactions occurring within the detection zones at various immunoassay steps, where the reduction of silver ions on gold nanoparticle-conjugated antibodies produces a detectable signal. This signal can be quantified using low-cost optical devices or visually examined. (c) Fluid Manipulation: The schematic demonstrates a passive reagent delivery system that operates without moving parts. Preloaded reagents flow over detection zones coated with capture proteins, facilitated by a vacuum generated by a disposable syringe. (d) Detection and Results: The images shows silver-enhanced signals on detection zones with HIV and syphilis antigens, and antibodies to goat IgG as a positive reference, illustrating clear distinctions between positive and negative samples. (e) Potential Applications: The diagram emphasizes the device’s applicability in diagnosing HIV and syphilis, showcasing its potential to revolutionize point-of-care diagnostics by providing a low-cost, efficient, and reliable testing method suitable for resource-limited settings. These features, highlight the transformative impact of microfluidic chips in enhancing diagnostic capabilities for infectious diseases in the developing world (Chin et al., 2011).

#### 3.4.1 Steps for design process

##### 3.4.1.1 Purpose of the ELISA

The foundation of designing an ELISA microfluidic device lies in its intended purpose, which primarily bifurcates into disease diagnosis and research and development. For disease diagnosis, the device must be engineered to deliver high sensitivity and specificity to detect disease biomarkers accurately. Meanwhile, devices aimed at research and development prioritize flexibility and repeatability, enabling researchers to modify and adapt the assay to different investigational needs without compromising the precision of the results (Figure 9).

##### 3.4.1.2 Select ELISA type

The selection of the ELISA type is pivotal in tailoring the device for specific analytes and sensitivity requirements. Direct ELISAs provide a more straightforward and less sensitive approach, suitable for abundant target antigens. Indirect ELISAs offer enhanced sensitivity through an additional antibody-enzyme complex. Sandwich ELISAs are employed for their high specificity and sensitivity, utilizing two antibodies to accurately capture the antigen. Competitive ELISAs are preferred for detecting small antigens and can achieve high sensitivity by the competitive binding principle.

##### 3.4.1.3 Sample type and preparation

The design must be adaptable to different sample types, each with unique preparation needs. For blood, serum, or plasma, microfluidic device channels may incorporate features for on-chip separation of cells. Cell cultures necessitate on-chip lysis and possibly washing steps for sample preparation. Devices intended for environmental samples might need integrated pre-concentration or purification stages to process complex matrices and extract analytes effectively.

##### 3.4.1.4 Microfluidic design considerations

The microfluidic design is central to the performance of the ELISA device, with channel design impacting the flow dynamics and non-specific binding. The integration of sample preparation components streamlines the assay process, enhancing the efficiency of the system. Decisions on reagent storage and delivery–whether to store reagents on-chip or introduce them externally-are critical for determining the autonomy and user-friendliness of the device.

##### 3.4.1.5 Detection method

Choice of detection method is crucial, as it influences the sensitivity and complexity of the device. Colorimetric detection is the simplest but offers limited sensitivity, appropriate for high-concentration analytes. Fluorescence detection increases sensitivity through the use of fluorescent labels, though it necessitates the integration of a fluorescence detector. Electrochemical detection methods are highly sensitive and compatible with portable device formats, but they may require complex integration of electronic components.

##### 3.4.1.6 Read out and data analysis

The design must address whether the readout will be processed on-chip, which could increase the complexity of the device, or off-chip, potentially reducing device cost but requiring additional instrumentation. Furthermore, the decision between quantitative and qualitative analysis affects the type of data output; quantitative provides exact measurements, whereas qualitative offers a binary indication of analyte presence.

##### 3.4.1.7 Fabrication method

The choice of fabrication material impacts the functionality and cost-effectiveness of the final device. PDMS is a popular choice for biocompatibility and flexibility, albeit prone to absorbance issues with hydrophobic substances. Glass offers a chemically inert and non-absorptive surface but at a higher cost and increased fragility. Plastics like PMMA present a cost-effective, scalable alternative but may require advanced bonding techniques for device assembly.

##### 3.4.1.8 Validation

Validation is a multi-faceted process ensuring the ELISA microfluidic device preforms reliably. Analytical validation assesses accuracy, precision, sensitivity, and specificity. Clinical validation involves comparing device performance against established diagnostic standards using real-world clinical samples. Operational validation encompasses user experience to ascertain the device is user-friendly and preforms consistently under practical conditions, preparing it for successful real-world application.

## 4 Discussion

**FIGURE 8:**
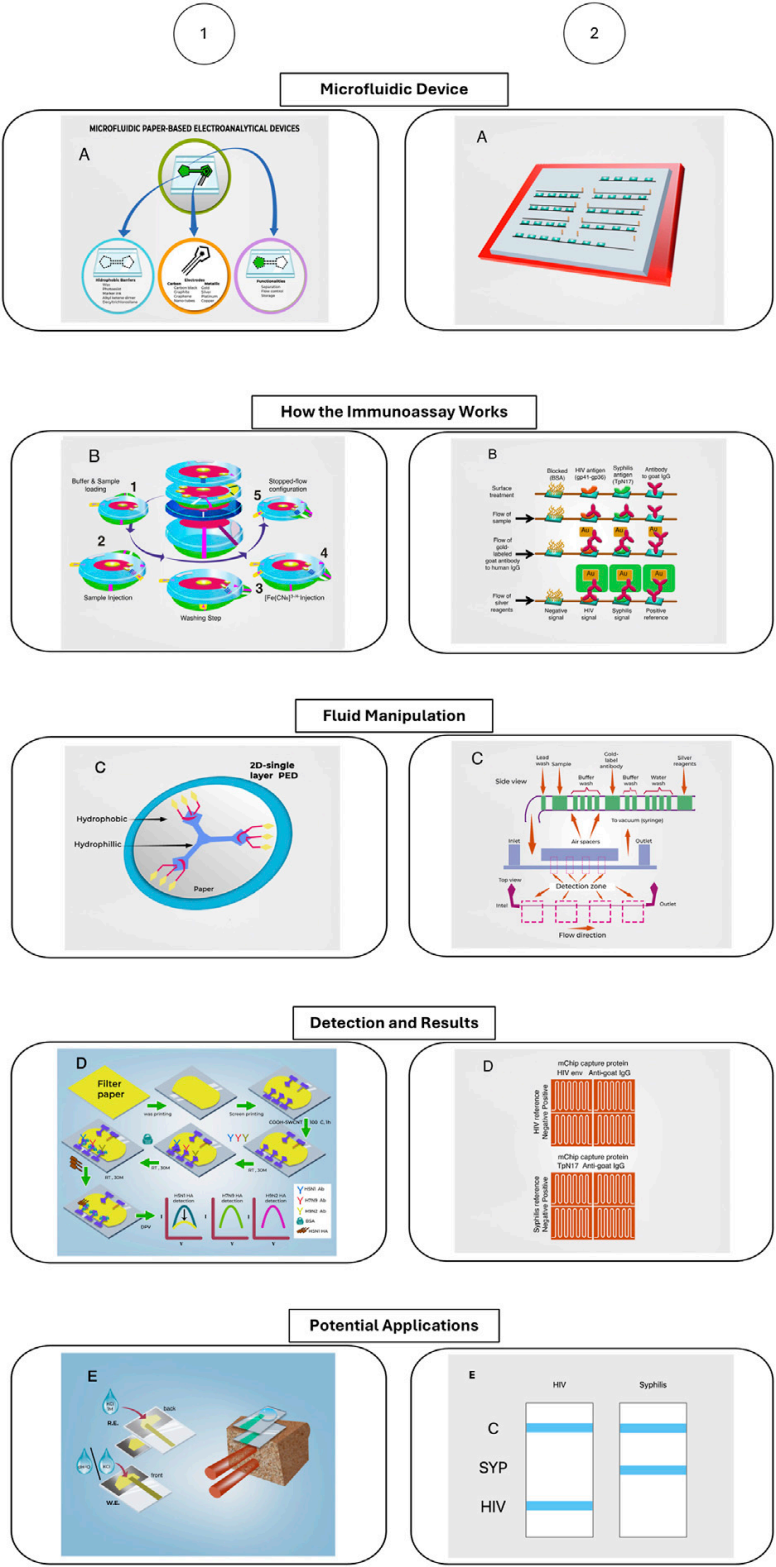
Advancement in electrochemical paper-based microfluidic devices. Modified from Chin et al. (2011), Holman et al. (2023). The figure depicts two different microfluidic devices, how the immunoassay the microfluidic device performs works, how the fluid manipulation in the device works, how the detection method and results are read, and the potential applications of these devices.

**FIGURE 9:**
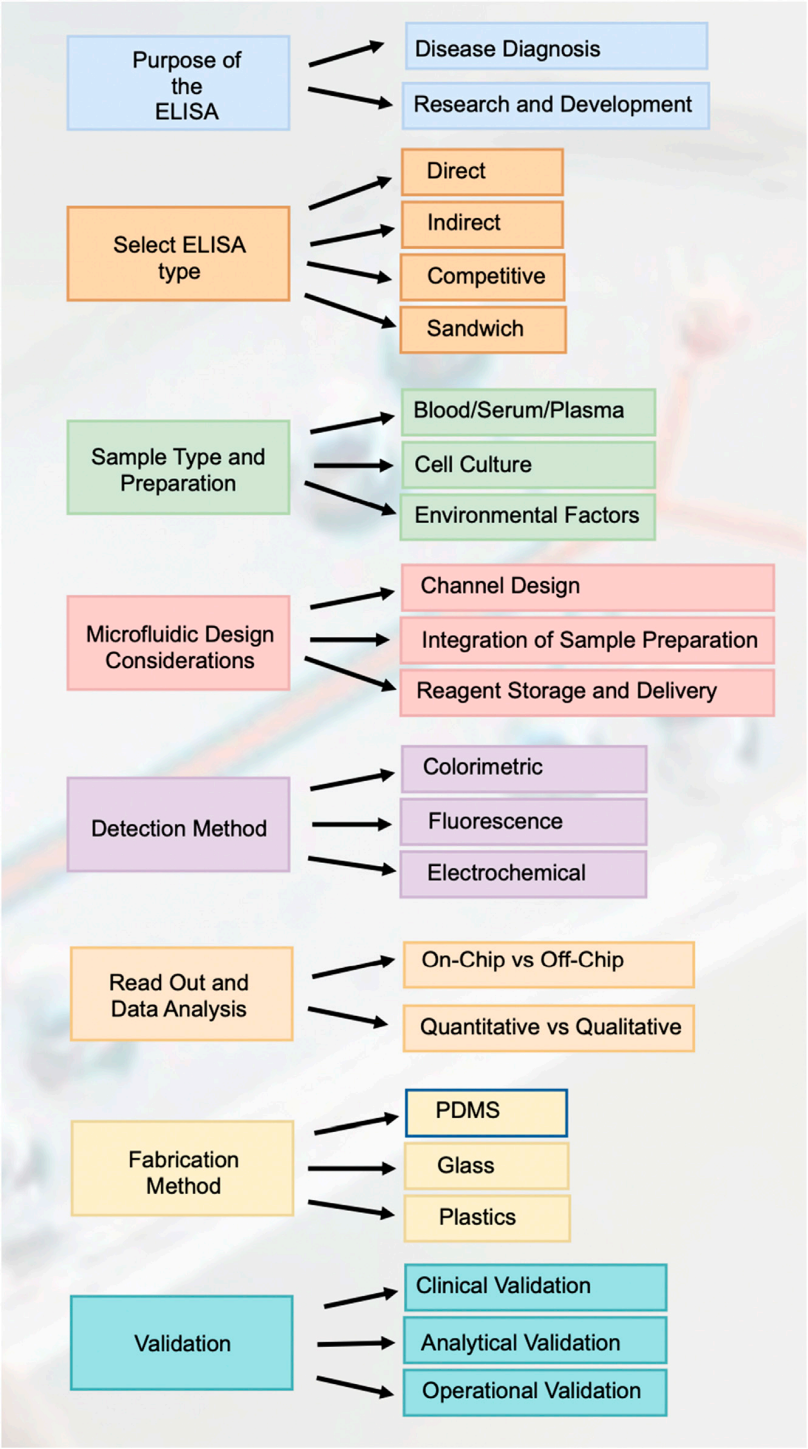
Road map for designing and implementing elisa in paper-based microfluidic devices. This figure outlines the comprehensive workflow for designing and implementing ELISA in paper-based microfluidic devices, covering each critical step from the purpose of the ELISA to validation.

Paper-based microfluidic devices show incredible potential as point-of-care diagnostics tools and represent a prime candidate to fulfil the World Health Organization’s REASSURED criteria for the key characteristics of any diagnostic test. The acronym stands for (Real-time connectivity, Ease of specimen collection, Affordable, Sensitive, Specific, User-friendly, Rapid, and robust, Equipment-free or simple, and Deliverable to end-users). The growing interest and development in recent decades of these systems has resulted in increased complexity of assays as shown in the Results section (Tables 1, 2; Figures 4–6). The miniaturization of techniques such as PCRMENDELEY CITATION PLACEHOLDER 0 and CRISPRMENDELEY CITATION PLACEHOLDER 1 exemplify the transition from laborious, costly and laboratory-reserved diagnostics to truly field deployable systems, ushering a new era for low-cost, reliable and accessible diagnostic tools for low-resource settings. Our research reveals that even though the process of fabrication and implementation of these devices can be very specific, as evidenced by the 32 different benchtop systems used to validate the µPADs performance (Supplementary Table S3; Figures 4, 6, 8, 9), a single device can serve multiple applications due to their adaptability of handling different reagents and biological samples.

Although µPADs have this incredible potential and diverse applications, there are still several important limitations that must be addressed for them to be commercially available and serve as a primordial POC diagnostic tool. These limitations include the difficulty of sample retention and evaporation during transportation, the variation that can arise in terms of specificity and sensitivity which can lead to false positive or negative results and the reagents used for the assays such as antibodies, enzymes, biological fluids must be able to withstand the transport to the point of application (Sher et al., 2017). The importance of addressing these limitations of the field lies on the prospect of mass production and commercially available testing kits, and although there has been enormous growth and progress towards these goals as evidenced by the results found in this review, there still is a lack of rapid processing and fabrication of high throughput without the need to use expensive reagents and/or specialized equipment to produce these systems. The need also exists for the µPADs ability to perform complex sequential reactions with high sensitivity and specificity but with a simple detection method, such as colorimetric based detection. We believe that this can be achieved through the integration of different techniques, such as employing a 3D device which allows for sequential flow mobility and in turn sequential reactions, allowing for more complex assay schemes in the same device. Also, given that the trend for smartphones has been increased availability and reduced cost, paired with better digital image cameras, the integration of smartphone-based optical detection method could allow for the desired complexity of assays without the need of specialized and costly equipment for the final signal-readout, as evidenced by MENDELEY CITATION PLACEHOLDER 2.

Prior to this systematic review, the potential of paper-based microfluidic devices (µPADs) in transforming diagnostics was recognized but not fully quantified or assessed across a range of applications. The literature hinted at the burgeoning capacity of these devices to meet the REASSURED criteria established by the World Health Organization, but a cohesive and comprehensive understanding was lacking. While the initial developments suggested that µPADs could serve as viable tools for point-of-care diagnostics, the nuances of their real-world applications, limitations, and adaptability remained under-explored.

With this review, we’ve cast light on the considerable advancements made in recent decades, which have seen µPADs evolve from concept to increasingly complex systems capable of conducting sophisticated assays such as PCR, ELISA, and CRISPR. The present knowledge, as synthesized from the reviewed studies, demonstrates the capability of µPADs to pivot from benchtop-bound procedures to portable, field-deployable systems. Through detailed meta-analysis, this review has revealed the promise of µPADs in democratizing access to diagnostics in low-resource settings, affirming their alignment with the REASSURED criteria—particularly in terms of affordability, ease of use, and the delivery of results.

Figure 8 provides an insightful illustration of the advancements in electrochemical paper-based microfluidic devices (μPEDs), showcasing their innovative features and applications. The diagram highlights the intricate design and functionality of these devices, emphasizing their role in enhancing diagnostic capabilities. It details key components and processes, such as the Microfluidic Device, which includes a sophisticated design capable of accommodating multiple samples with integrated reagent-loaded tubes. The ‘How the Immunoassay Works’ section outlines the biochemical reactions within the detection zones, demonstrating how the reduction of silver ions on gold nanoparticle-conjugated antibodies produces detectable signals, which can be quantified using low-cost optical devices or visually examined. The ‘Fluid Manipulation’ schematic demonstrates the precise control of fluid through hydrophobic barriers, ensuring accurate sample delivery. The ‘Detection and Results’ segment showcases the device’s capability to clearly distinguish between positive and negative samples, illustrated through the detection of HIV and syphilis antigens. Finally, the ‘Potential Applications’ section emphasizes the versatility of μPEDs in environmental monitoring, particularly in detecting chloride contamination. These features collectively underscore the transformative potential of μPEDs in creating low-cost, disposable, and efficient diagnostic tools suitable for point-of-care settings, particularly in resource-limited environments.

The importance of this subject is underscored by the pressing need for equitable health solutions worldwide. As this review has shown, µPADs stand at the forefront of this challenge, offering a pathway to accessible, reliable diagnostics that transcend the barriers imposed by traditional laboratory settings. Questions surrounding the efficacy, reliability, and applicability of µPADs for various diagnostics have been substantially answered, demonstrating that these devices can achieve high sensitivity and specificity while remaining cost-effective and user-friendly.

Nevertheless, important questions persist. The issue of sample retention and evaporation, the variability of specificity and sensitivity, and the robustness of reagents during transport are still unresolved challenges that this review has brought to light. These hurdles need to be surmounted to enable the mass production of µPADs and their establishment as primary tools for point-of-care diagnostics.

This review has mapped the trajectory of µPADs from theoretical constructs to practical diagnostic devices. It has illuminated the path traversed thus far and shed light on the journey ahead, charting a course toward a future where point-of-care diagnostics are accessible to all. While significant strides have been made, the quest for an optimal µPAD—characterized by seamless functionality, impeccable accuracy, and unparalleled ease of use—continues. The pursuit of such a device is not merely academic; it is a crucial endeavour with the potential to reshape global health landscapes.

## 5 Conclusions, limitations and future work

This systematic review and meta-analysis provide a comprehensive evaluation of paper-based microfluidic devices, particularly focusing on their applications in immunoassays. These devices offer significant advancements, such as low-cost, portability, and ease of use, making them particularly suitable for point-of-care diagnostics, especially in resource-limited settings (Heidari-Bafroui et al., 2023), as demonstrated by the integration of microfluidic channels and detection systems into a portable device capable of performing complex immunoassays with minimal user intervention and high sensitivity. The analysis highlights the versatility of paper-based microfluidics in various applications, from clinical diagnostics (Mumtaz et al., 2023), such as the detection of infectious diseases like malaria using simple colorimetric assays, and environmental monitoring (Sinha et al., 2022b), exemplified by the use of μPADs for detecting water, soil, or air contaminants. Additionally, these devices are used for drug delivery (Deng and Jiang, 2019) and nucleic acid amplification tests, demonstrated by integrated paper-based platforms for performing isothermal amplification of HIV DNA and detecting Influenza A (H1N1) from clinical specimens, (Basiri et al., 2021).

The advancements in fabrication techniques, including photolithography, laser cutting, inkjet printing, wax printing, and soft lithography, have enabled the development of intricate and highly functional microfluidic devices, as demonstrated by the creation of microchips that integrate various functions such as nucleic acid purification, DNA amplification, and signal detection, which have significantly improved the efficiency and sensitivity of diagnostic assays for immune-mediated diseases, (Menegatti et al., 2013). The integration of detection methods such as colorimetric, electrochemical, fluorescence, and spectroscopic techniques has further enhanced the capabilities of these devices (Jenkins et al., 2015), as evidenced by the development of printed silver nanoparticle ink electrodes on nitrocellulose with good conductivity, which can be used in electrochemical sensing for paper-based microfluidic devices.

Despite the significant advancements and potential demonstrated by paper-based microfluidic devices, several limitations need to be addressed to enhance their reliability and applicability in broader clinical settings (Rosenfeld and Bercovici, 2014). One major limitation is the variability in manufacturing quality, which can lead to inconsistent device performance and affect the reproducibility of results (Lepowsky et al., 2017), as demonstrated by the challenges faced in ensuring uniform reagent distribution across different batches of paper-based assays for urine analysis, leading to significant variations in test sensitivity and accuracy. The inconsistency underscores the need for standardized fabrication processes to ensure uniformity across batches of devices (Ulep et al., 2020), as evidenced by the development of a dual-layer paper microfluidic chip for detecting ROR1+ cancer cells, where variations in the wax printing process led to inconsistent capillary flow rates and detection sensitivity across different batches of device. Additionally, precise control of fluid flow within the microfluidic channels remains a challenge, impacting the accuracy of the assays. Achieving consistent fluid dynamics is critical for reliable diagnostics.

Another limitation is the integration of these devices with electronic systems for data processing and readout (Jenkins et al., 2015). While paper-based microfluidics offer simplicity and portability, the seamless incorporation of electronic components is still underdeveloped, limiting their real-time analytical capabilities (Novo et al., 2014). The long-term stability of reagents on paper substrates is also a concern, as degradation over time can compromise the accuracy and reliability of the devices (Zhu et al., 2018), as demonstrated by the reduced stability and sensitivity of physically adsorbed antibodies on cellulose, which desorb significantly during the washing steps, leading to decreased performance in paper-based ELISA tests. Moreover, while significant progress has been made in detection methods, achieving lower limits of detection with high sensitivity and specificity remains a crucial hurdle (Li et al., 2023). Lastly, the scalability of production while maintaining quality and affordability presents a significant challenge that must be overcome to facilitate widespread adoption (Sinha et al., 2022b).

Future research should focus on several key areas to address these limitations and advance the field of paper-based microfluidic devices. Developing advanced mechanisms for fluid control, such as micropumps, electro-osmotic flow, and enhanced capillary action, will be essential to improve assay precision and reliability (Kuan et al., 2016), as demonstrated by the microfluidic device integrating dual CMOS polysilicon nanowire sensors for on-chip whole blood processing and simultaneous detection of Hb and HbA1c, which significantly improved the mixing efficiency and uniform dilution of samples. Integrating these devices with digital technologies, including smartphones and portable electronic readers, can enable real-time data analysis and remote diagnosis, significantly enhancing their practical utility (Menegatti et al., 2013).

Implementing on-chip sample preparation steps, such as filtration, separation, and pre-concentration, can streamline workflows and improve the overall efficiency of the diagnostic process (Basiri et al., 2021). Future research should also include rigorous validation processes, encompassing cross-validation with established methods and extensive field testing, to ensure the robustness and reliability of these devices in various settings (Deng and Jiang, 2019). Lastly, incorporating aptamers as an alternative to antibodies in immunoassays can offer advantages such as higher stability and lower production costs, further enhancing the performance and applicability of these assays (Gandotra et al., 2023).

Good practices are critical in advancing the research and application of paper-based microfluidic devices. These practices include addressing limitations, data validation, and thorough evaluation of results, ensuring the reliability and accuracy of the devices and broadening their applicability across various fields, such as immunoassays, kinetics, and protein-ligand interactions (Lepowsky et al., 2017). To resolve current limitations, standardized manufacturing protocols should be developed to ensure consistent quality and performance, and advance fluid control mechanisms, such as micro pumps and electro-osmotic flow, should be incorporated to improve assay precision. Integration with electronic systems for real-time data analysis and remote diagnostics is essential, requiring flexible electronics and wireless communication technologies. Enhancing reagent stability through new immobilization methods and stabilizing agents and improving detection limits using nanoparticles and signal amplification techniques, can significantly enhance device performance (Basiri et al., 2021). Scalability of production can be achieved through mass production methods like roll-to-roll printing, ensuring quality and affordability. Rigorous data validation, including cross-validation with established methods and extensive field testing, is necessary to verify device accuracy. Comprehensive evaluation of results, including performance audits and user feedback, will identify areas for improvement, for example, better limits of detection, stability of reagents and manufacturing quality. Adopting these practices will address current limitations and propose solutions, ensuring the continued development and success of paper-based microfluidic devices.

## Data Availability

The original contributions presented in the study are included in the article/Supplementary Material, further inquiries can be directed to the corresponding authors.
