# Supplementary material for: Unveiling the state of the art: a systematic review and meta-analysis of paper-based microfluidic devices
Source: Front Bioeng Biotechnol. 2024 Aug 21;12:1421831. doi: 10.3389/fbioe.2024.1421831 (PMC11372461; doi:10.3389/fbioe.2024.1421831)
Supplement: Supplementary file 1 [file Table1.docx]

**Table S1. Overview of the microfluidic device articles with quality assessment.**

| Authors and year of publication | Setting | Total quality (%) | Microfluidic device and assay | Applications |
| --- | --- | --- | --- | --- |
| Pereira et al., 2024  (Pereira et al., 2024) | Portugal | 100 | Microfluidic Paper-Based Device Incorporated with Silica Nanoparticles for Iodide Quantification in Marine Source Dietary Supplements | Quantification of Iodide in seaweed and dietary supplements; Point-of-care; environmental analysis |
| Lee et al., 2023 (Lee et al., 2023) | China | 90 | Recent advancements in point-of-care testing of COVID-19 | Point-of-care |
| Pan et al., 2024  (Pan et al., 2024) | Netherlands | 100 | Paper-based microfluidic device for selective detection of peanut allergen Ara h1 applying black phosphorus-Au nanocomposites for signal amplification | Food safety testing; quality control in food manufacturing |
| Ko et al., 2023  (Ko et al., 2023) | China | 100 | Paper-based colorimetric sensors for point-of-care testing | Rapid point-of-care diagnosis; infectious disease diagnostic; pregnancy and fertility tests |
| Gandotra et al., 2023  (Gandotra et al., 2023) | Taiwan | 100 | A paper-based aptamer-sandwich assay for detection of HNP 1 as a biomarker for periprosthetic joint integrated microfluidic platform | Rapid diagnosis; point-of care; multiplexed assays; monitoring of treatment efficacy |
| Kumar et al., 2023  (Kumar et al., 2023) | India | 100 | Paper-based multiplex biosensors for inexpensive healthcare diagnostics: a comprehensive review | Multiplex detection in point-of-care testing; disease detection at an early stage |
| Sousa et al., 2023  (Sousa et al., 2023) | Germany | 100 | “Do it yourself” protocol to fabricate dual-detection paper-based analytical device for salivary biomarker analysis | Point of care; sensitive biomarker detection in saliva; diagnosis of periodontal disease |
| Heidari-Bafroui et al., 2023  (Heidari-Bafroui et al., 2023) | USA | 100 | Development of New Lab-on-Paper Microfluidics Platform Using Bi-Material Cantilever Actuators for ELISA on Paper | Point-of-care; environmental analysis; food safety testing; veterinary care |
| Jayachandran et al., 2024 (Jayachandran et al., 2024) | USA | 100 | Microfluidics-Based Blood Typing Devices: An In-Depth Overview | Immunoassay; blood type identification; rapid test protocols |
|  |  |  |  |  |
| Toldra et al., 2023  (Toldra et al., 2023) | Sweden | 100 | A 3D paper microfluidic device for enzyme-linked assays: Application to DNA analysis | Point-of-care; infectious disease diagnosis; environmental analysis |
| Kumar et al., 2023  (Kumar et al., 2023) | USA | 100 | Modeling of Paper-Based Bi-Material Cantilever Actuator for Microfluidic Biosensors | Point-of-care; environmental analysis; food safety |
| Mumtaz et al., 2023  (Mumtaz et al., 2023) | Pakistan | 100 | Prospects of Microfluidic Technology in Nucleic Acid Detection Approaches | Disease diagnosis; cell culture; chemical screening; drug delivery |
| Pinheiro et al., 2023  (Pinheiro et al., 2023) | Brazil | 100 | Low-Cost Microfluidic Systems for Detection of Neglected Tropical Diseases | Detection of malaria; diagnosis of protozoal diseases; viral disease detection |
| Li et al., 2023  (Li et al., 2023) | China | 80 | Microfluidics for COVID-19: From Current Work to Future Perspective | Rapid point-of-care; environmental analysis; food safety |
| Fu et al., 2023 (Fu et al., 2023) | USA | 100 | Paper-based All-in-One Origami Nanobiosensor for Point-of-Care Detection of Cardiac Protein Maters in Whole Blood | Multiplex detection of cardiac protein markers; rapid and sensitive testing for acute CvDs |
| Prat-Trunas et al., 2023  (Prat-Trunas et al., 2023) | Spain | 100 | Paper-based microfluidic electro-analytical device (PMED) for magneto-assay automation: Towards generic point-of-care diagnostic devices | Rapid point-of-care diagnostic tests of infectious diseases with high sensitive and quality responses |
| Wang et al., 2023  (Wang et al., 2023) | China | 100 | Electric yo-yo centrifugation combining with paper-based microfluidic immunoassay chip for inflammatory biomarkers detection in whole blood | Low cost and fast detection of inflammatory biomarkers |
| Holman et al., 2023  (Holman et al., 2023) | China | 100 | Advances on microfluidic paper-based electroanalytical devices | Biomarker detection; ion detection; pollution detection; heavy metal quantification; microorganism detection |
| Yousaf et al., 2023  (Yousaf et al., 2023) | Pakistan | 100 | Review on the prospects of microfluidic technology in nucleic acid detection approaches. | Nucleic acid amplification test; cancer diagnostic; drug delivery/testing; DNA sequencing; cell separation and treatment; cell culture and analysis; diagnostics |
| Ho et al., 2023  (Ho et al., 2023) | China | 100 | Parafilm®-based microfluidic devices by laser ablation and thermal bonding and no immunoassay was performed. | Point-of-care |
| Sinha et al., 2022  (Sinha et al., 2022) | India | 100 | Overall state of the paper-based microfluidic technology. | Healthcare diagnostics; environmental analysis; clinical diagnostics; signal-enhanced immunoassays; nucleic acid amplification test; blood plasma separation; cancer diagnostic; energy generation and storage |
| Lee et al., 2022  (Lee et al., 2022) | Korea | 100 | NPP (non-powered preconcentrator) lateral flow assay and (SARS-CoV-2) Antigen (Ag) lateral flow assay. | Clinical diagnostics; immunoassay; diagnostics |
| Singh et al., 2022  (Singh et al., 2022) | Korea | 80 | Review of glass-based microfluidic devices. | Point-of-care; cell culture and analysis; nucleic acid amplification test; drug delivery/testing; immunoassay |
| Biesalski et al., 2021  (Biesalski et al., 2021) | Germany | 80 | Paper-based lateral flow device, using lab-made cotton linter paper fibres (mostly cellulose) and modified with polymer hydrogels and protein-based assay. | Point-of-care; immunoassay |
| Aminian et al., 2021  (Aminian et al., 2021) | Iran | 60 | Review of paper-based analytical devices for blood grouping. | Point-of-care; blood plasma separation; healthcare diagnostics |
| Rezaei et al., 2021  (Rezaei et al., 2021) | Iran | 80 | Review on microfluidic devices for detection of RNA viruses. | Point-of-care; immunoassay; nucleic acid amplification test; healthcare diagnostics; clinical diagnostics |
| Ni et al., 2021  (Ni et al., 2021) | China | 100 | Microfluidic ruler-readout and CRISPR Cas12a-responded hydrogel-integrated paper-based analytical device (μReaCH-PAD) and invasive fungi (IF) assay. | Point-of-care; immunoassay; nucleic acid amplification test |
| Li et al., 2020  (Li et al., 2020) | China | 100 | Enzyme-encapsulated target-responsive DNA tetrahedra-cross-linked DNA, combined with a commercial glucose test strip to form a POCT device and DNA adenine methyltransferase assay (Dam assay) | Clinical diagnostics; nucleic acid amplification test |
| Yoon et al., 2020  (Yoon et al., 2020) | USA | 100 | Dual-layer paper microfluidic chip and cancer cell detection. | Cancer diagnostic |
| Bedin et al., 2019  (Bedin et al., 2019) | France | 100 | Glass‐fibre laser‐cut microfluidic device (paper‐based analytical device [PAD]) and chikungunya virus assay. | Point-of-care; clinical diagnostics; immunoassay |
| Deng and Jiang, 2019  (Deng and Jiang, 2019) | China | 100 | Review on advances in reagents storage and Release in self-contained point-of-care devices. | Clinical diagnostics; immunoassay; nucleic acid amplification test; storage; point-of-care; drug delivery/testing; DNA sequencing |
| Seth et al., 2018  (Seth et al., 2018) | Tanzania | 80 | Immunochromatographic thread‑based device in combination with nitrocellulose membrane and antigen-based assay. | Point-of-care; immunoassay |
| Wang et al., 2018  (Wang et al., 2018) | China | 100 | Microfluidic diatomite analytical devices (μDADs), consisting of highly porous photonic crystal bio silica channels and drug-based assay. | Point-of-care; pharmaceutical analysis |
| Song et al., 2017  (Song et al., 2017) | China | 100 | Integrated distance-based origami paper analytical device (ID-oPAD) and drug-based assay but the device presents universal applicability due to the use of aptamers. | Clinical diagnostics; environmental analysis |
| Hegener et al., 2017  (Hegener et al., 2017) | USA | 100 | Paper-based lateral flow microfluidic device and blood coagulation assay. | Point-of-care; blood plasma separation; clinical diagnostics; cell separation and treatment |
| Tasoglu et al., 2017  (Tasoglu et al., 2017) | USA | 100 | Review on paper-based microfluidic assays for urine analysis. | Clinical diagnostics; diagnostics; point-of-care; drug delivery/analysis |
| Packirisamy et al., 2016  (Packirisamy et al., 2016) | Canada | 80 | Review on microfluidic platforms for hormones detection. | Cell culture and analysis; cell separation and treatment; immunoassay; nucleic acid amplification test; point-of-care |
| Lin et al., 2016  (Lin et al., 2016) | China | 100 | Microfluidic Distance Readout Sweet Hydrogel Integrated Paper-based Analytical Device (µDiSH-PAD) and drug detection assay. | Drug delivery/analysis; environmental analysis; point-of-care; clinical diagnostics |
| Huang et al., 2016  (Huang et al., 2016) | Taiwan | 60 | Microfluidic device integrating dual CMOS polysilicon nanowire sensors for on-chip whole blood processing and blood assay. | Point-of-care; clinical diagnostics; healthcare diagnostics; blood plasma separation; protein detection |
| Jensen et al., 2016  (Jensen et al., 2016) | USA | 100 | Cellulose chromatography paper integrated with hydrogel microfluidic device and malaria detection assay. | Blood plasma separation; clinical diagnostics; point-of-care; immunoassay; cell separation and treatment; healthcare diagnostics |
| Yang et al., 2016  (Yang et al., 2016) | USA | 100 | Review on distance-based microfluidic quantitative detection methods for point-of-care testing. | Clinical diagnostics; environmental analysis; point-of-care; blood plasma separation; protein detection |
| Duman et al., 2016  (Duman et al., 2016) | Turkey | 100 | Review on rapid and alternative fabrication methods for microfluidic paper-based analytical devices. | Point-of-care |
| Lin et al., 2015  (Lin et al., 2015) | China | 100 | Target-Responsive DNA hydrogel mediated “Stop-Flow” microfluidic paper-based analytic device and drug detection, adenosine and metal ions assay. | Point-of-care; drug delivery/testing; immunoassay |
| Abbas et al., 2015  (Abbas et al., 2015) | USA | 60 | Review on Paper-based chemical and biological sensors: Engineering aspects. | Food safety monitoring; point-of-care; diagnostics |
| Niedl and Beta, 2015  (Niedl and Beta, 2015) | Germany | 100 | Munktell filter paper microfluidic device integrated with hydrogels and antibody-based assay. | Point-of-care |
| Rosenfeld and Bercovici, 2014  (Rosenfeld and Bercovici, 2014) | Israel | 100 | Cellulose filter paper microfluidic device and isotachophoresis-based assay. | Diagnostics |
| Jenkins et al., 2014  (Jenkins et al., 2014) | China | 60 | Review on printed electronics integrated with paper-based microfluidics and new methodologies for next-generation health care. | Point-of-care; healthcare diagnostics; clinical diagnostics |
| Conde et al., 2014  (Conde et al., 2014) | Portugal | 100 | Integrated capillary microfluidics with a microfabricated photodiode array and electronic instrumentation into a hand-held unit. | Point-of-care; immunoassay |
| Messina et al., 2014  (Messina et al., 2014) | Argentina | 60 | Silica nanoparticle-based microfluidic immunosensor with laser-induced fluorescence detection for the quantification of immunoreactive trypsin | Immunoassay; blood plasma separation; clinical diagnostics |
| Menegatti et al., 2013  (Menegatti et al., 2013) | Italy | 60 | Review on emerging analytical platforms for immune-mediated diseases (Lab-on-a-chip) | Immunoassay; nucleic acid amplification test; protein detection; cell culture and analysis; DNA sequencing; healthcare diagnostics; clinical diagnostics |
| Park et al., 2013  (Park et al., 2013) | USA | 100 | Cellulose chromatography paper and antibody-based assay. | Clinical diagnostics |
| Lin et al., 2011  (Lin et al., 2011) | China | 100 | PDMS, poly(ethylene glycol) diacrylate hydrogel integrated microfluidic device and protein and glucose-based assays. | Point-of-care; immunoassay |
| Huck et al., 2010  (Huck et al., 2010) | Germany | 100 | PDMS microfluidic device for droplet trapping, incubation, and release for enzymatic and cell-based assays | Cell culture and analysis; drug delivery/testing; DNA sequencing; nucleic acid amplification test |
| Hollfelder et al., 2008  (Hollfelder et al., 2008) | UK | 100 | Review on microdroplets in microfluidics | Cell separation and treatment; cell culture and analysis; immunoassay |
| Bhattacharyya and Klapperich, 2007  (Bhattacharyya and Klapperich, 2007) | USA | 100 | Plastic microfluidic device and chemiluminescent immunoassay for disease biomarkers in human serum samples | Point-of-care; self-diagnostic test; immunoassay |

**Table S2. Overview of the included articles based on their type of microfluidic device, detection method and validation.**

| Authors and year of publication | Country | Device(s) | Detection Method | Validation |
| --- | --- | --- | --- | --- |
| Pereira et al., 2024  (Pereira et al., 2024) | Portugal | Whatman Grade 4 filter paper; silica nanoparticles | Color intensity analysis | Comparison between potentiometric method and microfluidic device |
| Lee et al., 2023 (Lee et al., 2023) | China | Polydimethylsiloxane, paper, hydrogels, glass, silicon | Fluorescence, Surface-Enhanced Raman Scattering, Colorimetric detection, electrochemical detection | Comparison between gold standard and microfluidic device |
| Pan et al., 2024  (Pan et al., 2024) | Netherlands | Paper; Black Phosphorus (BP) Nanosheets; Gold (Au) Nanoparticles | Electrochemical sensing | Comparison between ELISA kit and microfluidic device. |
| Ko et al., 2023  (Ko et al., 2023) | China | Whatman grade 1; Whatman grade 3; Whatman grade 4 cellulose paper | Colorimetric analysis, signal generation | Image processing algorithms and spectrophotometry c analysis |
| Gandotra et al., 2023  (Gandotra et al., 2023) | Taiwan | Paper; Nitrocellulose (NC) membrane | ELISA with aptamers, fluorescence detection | Dilution curve development, gold-standard ELISA kit. |
| Kumar et al., 2023  (Kumar et al., 2023) | USA | Whatman Grade 41 Filter paper | Hygroexpansion-induced actuation | Mathematical model comparison with experimental results |
| Sousa et al., 2023  (Sousa et al., 2023) | Germany | Whatman filter paper grade 1, vegetal paper 3D pen resin | Colorimetric detection, electrochemical detection | Comparison between values found in literature and microfluidic device |
| Heidari-Bafroui et al., 2023  (Heidari-Bafroui et al., 2023) | USA | Whatman filter paper grade 41 | ELISA, Optical-color | Control experiment |
| Jayachandran et al., 2024 (Jayachandran et al., 2024) | USA | Whatman, Kleenex paper, paper towel, eucalyptus | Visual detection, image processing results | Comparison of various paper based blood typing platforms |
| Toldra et al., 2023  (Toldra et al., 2023) | Sweden | Whatman Grade 1 filter paper | ELISA, Optical-color | Comparison between benchtop ELISA and microfluidic system |
| Kumar et al., 2023  (Kumar et al., 2023) | India | Chromatography Whatman filter paper grade 1, nitrocellulose membrane | Colorimetric detection, fluorescence studies | Comparison between the detection of many target molecules on a one device and the microfluidic device |
| Mumtaz et al., 2023  (Mumtaz et al., 2023) | Pakistan | Glass, silicon | Optical-color, CRISPR, fluorescence-based detection | Comparison with existing research |
| Pinheiro et al., 2023  (Pinheiro et al., 2023) | Brazil | Glass, silicon, poly(dimethylsiloxane) | ELISA, PCR, LAMP, electrochemical detection, Optical-color | Comparison between conventional ELISA and PCR and microfluidic system |
| Li et al., 2023  (Li et al., 2023) | China | Glass, silicon, polymer | PCR, nucleic acid detection method, CRISPR, isothermal amplification technology | Comparison with the traditional rapid PCR instrument |
| Fu et al., 2023 (Fu et al., 2023) | USA | Whatman chromatography cellulose paper grade 1 | Electrochemical impedance spectroscopy | Tested of spiked human blood samples |
| Prat-Trunas et al., 2023  (Prat-Trunas et al., 2023) | Spain | Paper; carboxylic acid | PMED-based magneto immunoassay | Comparison with previous results in literature |
| Wang et al., 2023  (Wang et al., 2023) | China | Chromatography Whatman filter paper grade 1 | Calibration curves of ELISA analysis | Comparison with the whole blood samples tested in reference |
| Holman et al., 2023  (Holman et al., 2023) | China | Paper, glass, and inorganic materials | Optical-color, PCR, ELISA, isothermal amplification, CRISPR, aptamer-based detection, electrochemical, chemiluminescence, magnetic, SPR-based | Comparison between benchtop and microfluidic system |
| Ho et al., 2023  (Ho et al., 2023) | China | Parafilm-based | Scanning electron microscopy and fluorescence | Mechanical properties, actuators and biocompatibility |
| Sinha et al., 2022  (Sinha et al., 2022) | India | Paper | ELISA, electrochemical, optical-color and analytical methods | Comparison between benchtop and microfluidic system |
| Lee et al., 2022  (Lee et al., 2022) | Korea | Whatman paper | Optical-color | Control experiment and comparison between benchtop and microfluidic system |
| Singh et al., 2022  (Singh et al., 2022) | Korea | Silicon, elastomer, thermoset, PDMS, polycarbonate, hydrogel, paper and polyimide | Optical-color, optical-distance, electrochemical, mass spectrometry, surface enhanced Raman spectroscopy, microfluidic phase change method | Comparison between benchtop and microfluidic system |
| Biesalski et al., 2021  (Biesalski et al., 2021) | Germany | Cellulose with polymer hydrogels | Fluorescence | Comparison between benchtop and microfluidic system |
| Ni et al., 2021  (Ni et al., 2021) | China | Whatman No.1 filter paper | Optical-distance | Comparison between benchtop and microfluidic system |
| Li et al., 2020  (Li et al., 2020) | China | Cellulose with DNA tetrahedra-cross-linked hydrogel | Personal glucose meter | Control experiment and comparison between benchtop and microfluidic system |
| Yoon et al., 2020  (Yoon et al., 2020) | USA | Glass-fiber discs and cellulose chromatography paper | Smartphone fluorescence imaging | Comparison between benchtop and microfluidic system |
| Bedin et al., 2019  (Bedin et al., 2019) | France | Glass-fiber based paper | Optical-color and specific detection signal using the ImageJ software | Comparison between benchtop and microfluidic system |
| Deng and Jiang, 2019  (Deng and Jiang, 2019) | China | Cellulose with polymer hydrogels, nitrocellulose membrane, paper, hydrogel and glass-fiber based paper | Loop-mediated iso-thermal amplification, fluorescence, reverse transcription polymerase chain reaction, potentiometric sensor, electrochemical, aptamer-based detection, optical-color, optical-distance and smartphone integration | Comparison between benchtop and microfluidic system |
| Seth et al., 2018  (Seth et al., 2018) | Tanzania | Glass-fiber discs and nitrocellulose membrane | Optical-color | Control experiment and comparison between benchtop and microfluidic system |
| Wang et al., 2018  (Wang et al., 2018) | China | Microfluidic diatomite analytical device | Surface enhanced Raman spectroscopy | Comparison between benchtop and microfluidic system |
| Song et al., 2017  (Song et al., 2017) | China | Whatman No.1 filter paper | Optical-distance | Control experiment and comparison between benchtop and microfluidic system |
| Hegener et al., 2017  (Hegener et al., 2017) | USA | Glass-fiber based paper, nitrocellulose membrane, cellulose wicking pad and plastic backing card | Optical-distance and digital image analysis | Control experiment and comparison between benchtop and microfluidic system |
| Tasoglu et al., 2017  (Tasoglu et al., 2017) | USA | Cellulose chromatography paper, glass-fiber based paper and nitrocellulose membrane | Optical-color, electrochemical, fluorescence, smartphone integration and digital image analysis | Control experiment and comparison between benchtop and microfluidic system |
| Packirisamy et al., 2016  (Packirisamy et al., 2016) | Canada | Silica-on-silicon waveguide and PDMS | Laser-induced fluorescence, electrophoretic analysis, ELISA, electrochemical, high performance liquid chromatography, mass spectroscopy, fluorescence and spectrophotometer | Comparison between benchtop and microfluidic system |
| Lin et al., 2016  (Lin et al., 2016) | China | “Sweet” Hydrogel and Whatman No.1 filter paper | Optical-color, optical-distance and digital image analysis | Control experiment and comparison between benchtop and microfluidic system |
| Jensen et al., 2016  (Jensen et al., 2016) | USA | Cellulose chromatography paper and hydrogel | Optical-color and digital image analysis | Control experiment and comparison between benchtop and microfluidic system |
| Yang et al., 2016  (Yang et al., 2016) | USA | Glass, paper, silicon, thread and PDMS | Optical-distance, optical-color, fluorescence and ELISA | Comparison between benchtop and microfluidic system |
| Duman et al., 2016  (Duman et al., 2016) | Turkey | Whatman paper | Optical-color | Comparison between benchtop and microfluidic system |
| Lin et al., 2015  (Lin et al., 2015) | China | Whatman No.1 filter paper and DNA hydrogel | Optical-color and aptamer-based | Control experiment |
| Niedl and Beta, 2015  (Niedl and Beta, 2015) | Germany | Cellulose filter paper and hydrogel | Optical-color | Control experiment |
| Rosenfeld and Bercovici, 2014  (Rosenfeld and Bercovici, 2014) | Israel | Cellulose filter paper | Fluorescence | Comparison between benchtop and microfluidic system |
| Conde et al., 2014  (Conde et al., 2014) | Portugal | PDMS and a-Si:H photodiodes | Chemiluminescence ELISA and optical-photodiodes | Comparison between benchtop and microfluidic system |
| Park et al., 2013  (Park et al., 2013) | USA | Cellulose chromatography paper | Optical-color | Comparison between benchtop and microfluidic system |
| Lin et al., 2011  (Lin et al., 2011) | China | PDMS, poly(ethylene glycol) diacrylate hydrogel and glass | Optical-color | Control experiment and comparison between benchtop and microfluidic system |
| Huck et al., 2010  (Huck et al., 2010) | Germany | PDMS | Fluorescence, surface enhanced Raman spectroscopy, polymerase chain reaction, laser-induced fluorescence spectroscopy, mass spectrometry method, electrophoretic analysis, high performance liquid chromatography, mass spectroscopy, electrochemical and ELISA | Control experiment and comparison between benchtop and microfluidic system |
| Hollfelder et al., 2008  (Hollfelder et al., 2008) | UK | PDMS | Fluorescent camera, mercury-arc lamp, laser-induced fluorescence spectroscopy and photo-multiplier tube | Control experiment and comparison between benchtop and microfluidic system |
| Bhattacharyya and Klapperich, 2007  (Bhattacharyya and Klapperich, 2007) | USA | Plastic | Chemiluminescence and instant photographic film | Comparison between benchtop and microfluidic system |

**Table S3. Meta-analysis continued: Mobility and Benchtop system**

| Meta-Analysis cont. | | |  |
| --- | --- | --- | --- |
| **Author, Year, Country** | **Mobility** | **Benchtop system** |  |
|  |  |  |  |
| Pereira et al., 2024, Portugal | CA/E-O/PP/SP/MiP | SS/SPM/HPLC-MS/GC-MS |  |
| Lee et al., 2023, China | E-O/CA/AF | PCR/ELISA/HPLC-MS |  |
| Pan et al., 2024, Netherlands | CA/E-O/ MiP/PP/SP/SF/CP/AF/SeF/PF/SegF/FFD | SS/B-M |  |
| Ko et al., 2023 China | SegF/FFD | B-M |  |
| Gandotra et al., 2023, Taiwan | CA/MiP | B-M |  |
| Kumar et al., 2023, USA | CA/E-O | SS/FC |  |
| Sousa et al., 2023, Germany | CF | LS-MS |  |
| Heidari-Bafroui et al., 2023, USA | AF | B-M |  |
| Jayachandran et al., 2024 , USA | CA | DIA/SA/SEM |  |
| Toldra et al., 2023 , Sweden | AF/CA | SPM/FC/TC/SI |  |
| Kumar et al., 2023, USA | SegF | B-M |  |
| Mumtaz et al., 2023, Pakistan | CA/E-O/PP/MiP/SP | B-M |  |
| Pinheiro et al., 2023, Brazil | E-O/CF/PP | B-M/FC/HPLC-MS/GC-MS |  |
| Li et al., 2023, China | SegF | B-M/ELISA/PCR |  |
| Fu et al., 2023, USA | CF | B-M/DIA |  |
| Prat-Trunas et al., 2023, Spain | CF/CP | B-M/CFR |  |
| Wang et al., 2023, China | CF | B-M/SS/UP |  |
| Holman et al., 2023, Pakistan | CA/CF | NA |  |
| Ho et al., 2023, China | ParMP | CPL |  |
| Sinha et al., 2022, India | CA | NA |  |
| Lee et al., 2022, Korea | CA | COVID-Ag |  |
| Singh et al., 2022, Korea | E-O/CF/CA | NA |  |
| Biesalski et al., 2021, Germany | CA | UP |  |
| Ni et al., 2021, China | CA | qRT-PCR |  |
| Li et al., 2020, China | CA | PGM |  |
| Yoon et al., 2020, USA | CA | FM/SFM |  |
| Bedin et al., 2019, France | CA | MAC-ELISA |  |
| Jiang et al., 2019, China | CF/CA/PP | PGM/PTT |  |
| Seth et al., 2018, Tanzania | CA | CLFA |  |
| Wang et al., 2018, China | CA | NDCP |  |
| Song et al., 2017, China | CA | PGM |  |
| Hegener et al., 2017, USA | CA | C-PT-INR |  |
| Tasoglu et al., 2017, USA | CA/CF | CUTT/Urisys 1100®/cobas® ] |  |
| Packirisamy et al., 2016, Canada | CA/MiP | CFR |  |
| Lin et al., 2016, China | CA | LC-MS/GC/GC-MS |  |
| Jensen et al., 2016, USA | SP/CA | SCP |  |
| Yang et al., 2016, China | CA | NS |  |
| Duman et al., 2016, Turkey | CA | RUT |  |
| Lin et al., 2015, China | CA/SF | NS |  |
| Beta et al., 2015, Germany | CA | BC |  |
| Bercovici et al., 2014, Israel | CA/E-O | IEGM |  |
| Conde et al., 2014, Portugal | CA/CP/AF/SeF | PiA/MCR/L-iA/LBM |  |
| Park et al., 2013, USA | CA | SS |  |
| Lin et al., 2011, China | CA | FM/DIA |  |
| Huck et al., 2010, Germany | CF/SegF/PF | TC/FC/HPLC |  |
| Hollfelder et al. 2008, UK | FFD/SP/CF | FLC/MAL/LIFS/PMT |  |
| Bhattacharyya et al., 2007, USA | SP | ELISA |  |
|  |  |  |  |

**Table S4. Meta-analysis continued: Validation and Sensitivity and detection limits**

| Meta-Analysis cont. | | |  |
| --- | --- | --- | --- |
| **Author, Year, Country** | **Validation** | **Sensitivity and detection limits** |  |
|  |  |  |  |
| Pereira et al., 2024, Portugal | PCR/ELISA/CRISPR/IA/LAMP | 3.0 μM |  |
| Lee et al., 2023, China | Sen/Spe/LOD/B-M | NA |  |
| Pan et al., 2024, Netherlands | ELISA | 11.8 ng/mL |  |
| Ko et al., 2023 China | ELISA/COVID-AG/LC-MS/SFM | 200 genomic copies/ μL |  |
| Gandotra et al., 2023, Taiwan | A-BD/ ELISA | 0.5 mg/L |  |
| Kumar et al., 2023, USA | B-M/LOD/Sen/Spe | NA |  |
| Sousa et al., 2023, Germany | B-M/CE | 200 U/mL |  |
| Heidari-Bafroui et al., 2023, USA | LOD/Sen/Spe | 2.27 ng/mL |  |
| Jayachandran et al., 2024 , USA | ELISA | NA |  |
| Toldra et al., 2023 , Sweden | Sen/Spe/LOD/qRT-PCR/SeF/SF | 10 genome copies/ μL |  |
| Kumar et al., 2023, USA | LC-MS/ELISA | 136 pg/mL |  |
| Mumtaz et al., 2023, Pakistan | LOD/Sen/S-C/S-M | NA |  |
| Pinheiro et al., 2023, Brazil | CE/MP/BioC/Sen/LOD/Spe | NA |  |
| Li et al., 2023, China | SFM/LC-MS | 200 pg/mL |  |
| Fu et al., 2023, USA | SFM/BioC | 4.6 pg/mL |  |
| Prat-Trunas et al., 2023, Spain | ELISA/S-M | 12 ng/mL |  |
| Wang et al., 2023, China | Sen/LOD/CE | 580 pg/mL - 800 pg/mL |  |
| Holman et al., 2023, Pakistan | B-M | NA |  |
| Ho et al., 2023, China | MP/Ac/BioC | NA |  |
| Sinha et al., 2022, India | B-M | 5 cells |  |
| Lee et al., 2022, Korea | CE/B-M | >90% and Ct ≥ 30, |  |
| Singh et al., 2022, Korea | B-M | 50 zmol (3.6x10-9) / 10-4 RIU |  |
| Biesalski et al., 2021, Germany | B-M | NS |  |
| Ni et al., 2021, China | B-M | 10 CFU/mL |  |
| Li et al., 2020, China | CE/B-M | 0.001 U/mL |  |
| Yoon et al., 2020, USA | B-M | 0.1 cells/μL |  |
| Bedin et al., 2019, France | B-M | Sen=70.6% and Spe=98% |  |
| Jiang et al., 2019, China | B-M | 1 × 10^−18 M/ 0.1 ng/ml |  |
| Seth et al., 2018, Tanzania | CE/B-M | 30–300 ng/ml |  |
| Wang et al., 2018, China | B-M | 10 ppb |  |
| Song et al., 2017, China | CE/B-M | Cocaine: 1.8 μM / Adenosine: 20 μM |  |
| Hegener et al., 2017, USA | CE/B-M | NS |  |
| Tasoglu et al., 2017, USA | CE/B-M | NS |  |
| Packirisamy et al., 2016, Canada | B-M | 0.1–10 μIU mL-1/ 1-5 ng/mL /240 ng/ml / 25 ng/ml |  |
| Lin et al., 2016, China | CE/B-M | 3.8 µM - 5.8 µM |  |
| Jensen et al., 2016, USA | CE/B-M | 7.5 nM |  |
| Yang et al., 2016, China | B-M | See Table 1. in article |  |
| Duman et al., 2016, Turkey | B-M | 1 unit/mL |  |
| Lin et al., 2015, China | CE | Cocaine: 50 μM, Adenosine |  |
| Beta et al., 2015, Germany | CE | 10^4 bacteria |  |
| Bercovici et al., 2014, Israel | B-M | 10–100 bacteria copies/mL |  |
| Conde et al., 2014, Portugal | B-M | 2 nM / antibody-antigenaffinity constant |  |
| Park et al., 2013, USA | B-M | S-C |  |
| Lin et al., 2011, China | CE/B-M | BSA: 1–150 μM / Glucose: 50 mM-2M |  |
| Huck et al., 2010, Germany | CE/B-M | 500 μM / 1nM / S-C / S-M |  |
| Hollfelder et al. 2008, UK | CE/B-M | S-C |  |
| Bhattacharyya et al., 2007, USA | B-M | 0.1 mg/L High sensitivity |  |

Opt-C, optical-color; Opt-D, optical-distance; MS, mie scattering; SA, smartphone application; PGM, personal glucose meter; FL, fluorescent; EL, electrochemical; SEM, scanning electron microscopy; SFI, smartphone fluorescence imaging; SDS, specific detection signal; SERS, surface enhanced Raman spectroscopy; PCR, polymerase chain reaction; ELISA, enzyme linked immunosorbent assay; IA, isothermal amplification; CRISPR, clustered regulatory interspaced short palindromic repeats; A-BD, aptamer-based detection; Chemi; chemiluminescence; Mag, magnetic; SPR, surface plasmon resonance; AM, analytical methods; MaS, mass spectrometry method; PCM, microfluidic phase change method; LAMP, loop-mediated isothermal amplification; RT-PCR, reverse transcription polymerase chain reaction; PS, potentiometric sensor; LiFL, laser-induced fluorescence; ElpA, electrophoretic analysis; HPLC-MS, high performance liquid chromatography mass spectroscopy; SPM, spectrophotometer; SI, smartphone integration; DIA, digital image analysis; Mir, microwave irradiation; Chemi-ELISA, chemiluminescence ELISA; Opt-PHD, optical-photodiodes; LIFS, laser-induced fluorescence spectroscopy; FLC, fluorescent camera; MAL, mercury-arc lamp; PMT, photo-multiplier tube; IPF, instant photographic film; CA, capillary action; ParMP, parafilm micropump; CF, continuous flow; E-O, electro-osmotic flow; PP, peristaltic pump; MiP, micro pump; SP, syringe pump; SF, stop flow; CP, capillary pumps; AF, autonomous flow; SeF, sequential flow; PF, Poiseuille flow; SegF, segmented flow; FFD, flow focusing device; SS, spectrometric system; UP, uncoated paper; PGM, personal glucose meter; CPL, conventional photolithography; COVID-Ag; commercial COVID-19 Ag kit; qRT-PCR, quantitative reverse transcription polymerase chain reaction; FM, fluorescence microscope; SFM, smartphone fluorescence microscope; MAC-ELISA, IgM capture enzyme‐linked immunosorbent assay; PTT, pregnancy test strips; CFR, confocal reflectance microscopy; C-PT-INR, coagulometer, PT-INR; CUTT, commercial urine test strips; Urisys 1100®; cobas® u 411 analyzer; LAURA® Semi-Automated Urine Strip Reader; LC-MS, liquid chromatography-mass spectrometry; GC, gas chromatography; GC-MS, gas chromatography-mass spectrometry; SCP, standard centrifuge; RUT, rapid urease test; BC, bacterial contamination assays; IEGM, isotropically etched glass microchip; PiA, picoammeter; MCR, monochromator; L-iA, lock-in amplifier; LBM, light beam chopper; TC, thermocycler; FC, flow cytometer; B-M, comparison between benchtop and microfluidic system; CE, control experiment; MP, mechanical properties; Ac, actuators; BioC, biocompatibility; S-C, single-cell-level detection; NS, not specified; Sen, sensitivity; LOD, limit of detection; Spe, specificity; S-M, single-molecule-level detection

AA, antibody; PA, protein; DamA, Dam; DD, drug detection; AgLFA, antigen lateral flow assay; IFA, invasive fungi assay; CaA, cancer; NAD, nucleic acid detection; ImA, immunoassay; CeA, cellular; LEFA, lateral electrophoretic flow; HA, hormone; BCA, blood coagulation; UA, urine; EA, enzyme; AdA, adenosine; MIA, metal ion; IPA, isotachoporesis; GA, glcuose; CCP, cellulose chromatography paper; CCPH, cellulose with polymer hydrogels; CCP-DNAH, cellulose with DNA tetrahedra-cross-linked hidrogel; Par, Parafilm; WP, Whatman paper; WFP, Whatman No.1 filter paper; GF-D, GF/D glass fiber substrate; GFP, glass-fiber based paper; GFD, glass-fiber discs; NCM, nitrocellulose membrane; μDADs, microfluidic diatomite analytical device; Pap, paper; Gla, glass; IM, inorganic materials; Sil, silicon; Ela, elastomer; The, thermoset; PDMS, polydimethylsiloxane; Pol-C, polycarbonate; Hyd, hydrogel; Pol-I, polyimide; SOS, silica-on-silicon waveguide; CWP, cellulose wicking pad; PBC, plastic backing card; SHyd, “Sweet” hydrogel; Thr, thread; DNA-Hy, DNA hydrogel; CFP, cellulose filter paper; PHD, a-Si:H photodiodes; PEG-DA-Hyd, poly(ethylene glycol) diacrylate hydrogel; Pla, plastic; LC, laser cutting; NP, negative photoresist; LG, grafting; LWP, wax printing; LA, laser ablation; PL, photolithography; LE, embossing; LFP, flexographic printing; LIP, inkjet printing; LPDMS, PDMS screen printing; LPCT, printed circuit technology; LTS, two-dimensional shaping; LPT, plasma treatment; LFTAS, fast lithographic activation of sheets; LSPP, stacking of patterned paper; L3DWP, 3D wax printing; DrE, dry etching; ChE, chemical etching; TB, thermal bonding; AB, anodic bonding; LSP, screen printing; WeE, wet etching; LPP, pen plotting; SL, soft lithography; HE, hot-embossing; A-CD, clinical diagnostics; A-PA, pharmaceutical analysis; A-EA, environmental analysis; A-ImA, immunoassay; A-NAA, nucleic acid amplification test; A-BPS, blood plasma separation; A-D, diagnostic; A-S, storage; A-POC, point-of-care; A-CaD, cancer diagnostic; A-DD, drug delivery/testing; A-DNAS, DNA sequencing; A-CST, cell separation and treatment; A-CC, cell culture/analysis; A-HD, healthcare diagnostics; A-SEImA, signal-enhanced immunoassays; A-EGA, energy generation and storage; A-PD, protein detection; A-SDT, self-diagnostic test.
